# Estrogen receptor α (*Esr1*) mediates estrogen’s ability to promote papillomavirus-induced cutaneous disease in male and female mice

**DOI:** 10.1128/mbio.00783-26

**Published:** 2026-05-29

**Authors:** Sheikh A. Umar, Wei Wang, Donghwan Jeon, Lam Khue Pham, Ella T. Ward-Shaw, Denis Lee, Rong Hu, Andrea Bilger, Megan E. Spurgeon, Paul F. Lambert

**Affiliations:** 1 McArdle Laboratory for Cancer Research, Department of Oncology, University of Wisconsin School of Medicine and Public Health 219455 , Madison, Wisconsin, USA; 2 Department of Microbiology, Genetics, & Immunology, Michigan State University 220155, East Lansing, Michigan, USA; 3 Department of Pathology and Laboratory Medicine, University of Wisconsin School of Medicine and Public Health 189586, Madison, Wisconsin, USA; 4 University of Wisconsin Carbone Cancer Center, University of Wisconsin School of Medicine and Public Health 206022, Madison, Wisconsin, USA; 5 John W. and Jeanne M. Rowe Center for Research in Virology, Morgridge Institute for Research 145254, Madison, Wisconsin, USA; Duke University School of Medicine, Durham, North Carolina, USA

**Keywords:** MmuPV1, ERα, immune evasion, cutaneous infection, host-pathogen interaction

## Abstract

Persistent papillomavirus infection is a key driver of neoplastic progression in both mucosal and cutaneous epithelia. Although estrogen is a well-established cofactor in HPV-associated cervical carcinogenesis, its role in cutaneous papillomavirus disease has remained poorly understood. Using a murine papillomavirus type 1 (MmuPV1) infection model in immunocompetent *FVB/N* mice, we investigated whether estrogen influences viral pathogenesis in infected cutaneous epithelia. Estrogen treatment significantly increased wart incidence and growth independent of sex and was associated with pronounced epithelial hyperplasia, dysplasia, koilocytosis, and features of invasive squamous cell carcinoma. Systemically, estrogen reduces circulating leukocytes, while locally suppressing adaptive immune infiltration and enriching neutrophils within lesions, indicating broad immunosuppression coupled with selective innate enhancement. MmuPV1 E1^E4 and L1 RNA and protein levels were elevated in warts of estrogen-treated WT mice, indicative of enhanced productive infection. Importantly, *Esr1* germline knockout mice exhibited significantly reduced wart incidence and size in both males and females, lower MmuPV1 E1^E4 and L1 transcript levels, coupled with the absence of estrogen-induced depletion of circulating immune cells, demonstrating that ERα signaling mediates estrogen-driven epithelial proliferation, immune modulation, disease progression, and productive viral infection. Collectively, these novel findings indicate that estrogen is a potent cofactor in cutaneous papillomavirus disease, acting through ERα-dependent pathways to drive virally -induced neoplastic diseases, including cancer.

**IMPORTANCE:**

Papillomaviruses are major causes of cancer in both women and men, yet the factors that influence disease severity remain incompletely understood. While estrogen is known to cooperate with papillomaviruses to drive cervical cancer, its role in papillomavirus-associated skin disease has not been defined. Our study demonstrates that estrogen significantly amplifies papillomavirus-induced skin pathology in both male and female mice, increasing the frequency, size, and severity of epidermal lesions. These findings reveal a previously unrecognized hormonal influence on cutaneous papillomavirus disease and suggest that sex hormones may broadly modulate papillomavirus-associated cancers beyond the reproductive tract.

## INTRODUCTION

The *Papillomaviridae* family consists of small, non-enveloped viruses with circular double-stranded DNA genomes, typically ranging from seven to eight kilobase pairs. Papillomaviruses encode four core proteins: E1 and E2, which drive viral replication, and L1 and L2, which form the capsid. Human papillomaviruses (HPVs) also produce accessory proteins, E1^E4, E5, E6, E7, and E8^E2, which support the viral life cycle (1). Papillomaviruses are epitheliotropic viruses that infect stratified squamous epithelia and establish a life cycle tightly coupled with keratinocyte differentiation (2). While most infections are transient, persistent papillomavirus infection is a major risk factor for neoplasia at both mucosal and cutaneous sites (3). HPVs are etiologically linked to cervical cancer and other anogenital and oropharyngeal malignancies, and accumulating evidence implicates host factors, including hormonal signaling, in modulating the disease trajectory (4–6). Although high-risk HPV types are most strongly associated with anogenital and oropharyngeal cancers, several HPV types also cause cutaneous warts and contribute to a spectrum of skin neoplasias albeit with a substantially lower overall disease burden (7, 8). Nonetheless, cutaneous papillomaviruses provide valuable insight into fundamental host-virus interactions, including hormonal and immune mechanisms that may be conserved across papillomavirus infections. Estrogen, acting primarily through estrogen receptor alpha (ERα), has been shown to synergize with HPV oncogenes to promote epithelial proliferation and neoplasia of cervicovaginal epithelia (9). Continuous estrogen exposure is required for the initiation and maintenance of cervical cancer in HPV transgenic mouse models (10, 11). Estrogen withdrawal leads to tumor regression, underscoring its role as a critical cofactor in papillomavirus-driven carcinogenesis (12, 13). Beyond mucosal sites, the influence of estrogen on cutaneous papillomavirus infection remains poorly defined.

Murine papillomavirus type 1 (MmuPV1) provides a tractable *in vivo* model for studying papillomavirus pathogenesis in immunocompetent hosts, recapitulating key features of human diseases, including epithelial hyperplasia, koilocytosis, and progression to SCC-like invasive pathology under permissive conditions (14–21). Recent studies have highlighted that host immune competence and microenvironmental cues critically determine lesion persistence and severity (22). Estrogen is known to exert broad immunomodulatory effects, dampening adaptive immune responses while skewing innate immunity toward neutrophil-dominant inflammation (23, 24). Such changes may compromise antiviral surveillance and create a permissive niche for viral persistence and productive infections. Previous studies from our laboratory have demonstrated that estrogen exerts a profound influence on MmuPV1 infection within cervicovaginal tissues (14, 25). Recently, our laboratory reported that estrogen acts synergistically with keratin 17 to impair systemic immunity and remodel the tumor microenvironment, promoting viral persistence and driving neoplastic progression in MmuPV1-infected cervicovaginal epithelia (24). These studies underscore the dual role of estrogen in epithelial proliferation and immune modulation during papillomavirus pathogenesis. However, whether similar mechanisms operate in cutaneous infections, and in males as well as females, has not been addressed.

Here, we investigated the effect of estrogen on MmuPV1-induced cutaneous disease in *FVB/N* mice using pre- and post-estrogen (E2) treatment paradigms. Our findings revealed that estrogen promotes papillomavirus pathogenesis by enhancing epithelial proliferation and differentiation states conducive to viral replication, and attenuating systemic and local immune responses, facilitating lesion growth and productive infection. Estrogen acts as a potent cofactor in cutaneous papillomavirus disease independent of the timing of administration and sex. These results provide mechanistic insights into how hormonal signaling intersects with the epithelial and immune pathways that drive viral persistence and progression. Importantly, studies in *Esr1* germline knockout mice demonstrated that ERα signaling is essential for these effects, as its loss significantly reduces wart incidence and size, reduces viral gene transcripts within the warts, and prevents estrogen-induced immune alterations. These results confirmed that estrogen-driven epithelial proliferation, immune modulation, and disease severity are mediated through ERα-dependent pathways.

## MATERIALS AND METHODS

### Mouse keratinocyte culture, transfection with re-circularized MmuPV1 genome, and 17β-estradiol treatment

Primary mouse keratinocytes were cultured with feeders in a 3:1 mixture of Dulbecco’s modified Eagle medium, high glucose (Life Technologies, 31053028), and F12 medium (Thermo Fisher, 21041025), supplemented with 5% fetal bovine serum (Cytiva, SH30910.03) or 5% charcoal-stripped FBS when required. The medium additionally contained penicillin G (Invitrogen, 15140-023), adenine (Sigma, A9795), cholera toxin (ICN, 150005), epidermal growth factor (R&D Systems, 236-EG), insulin (Merck, 407709), and hydrocortisone (Calbiochem, 386698). Cells were maintained at 37°C with 5% CO₂ in a humidified incubator and grown to approximately 80% confluence. Keratinocytes isolated from an *FVB/N* mouse were plated in 24-well plates and transfected the following day with either 300 ng of pcDNA6-dsRed or 300 ng pcDNA6-dsRed plus 700 ng re-circularized MmuPV1 genome, using Lipofectamine 3000 according to the manufacturer’s instructions. pcDNA6-dsRed expresses dsRed and confers blasticidin resistance, enabling the selection of successfully transfected cells. At 48 h post-transfection, cells were placed under blasticidin selection (7 µg/mL) for 4 days, after which surviving cells were pooled and expanded. For estrogen-treatment experiments, selected keratinocytes were seeded into 6-well plates and cultured in DMEM/F12 medium containing 5% FBS until reaching ~80% confluence. Cells were then serum-starved overnight in charcoal-stripped FBS and treated the next day with 17β-estradiol (Sigma, E2758) at 10 nM for 4 h as previously (26). Following treatment, cells were lysed directly in TRIzol (Thermo Fisher, 15596026) and processed immediately for RNA isolation.

### RNA extraction, cDNA synthesis, and qPCR

Total RNA was extracted using the RNeasy Plus Mini Kit (Qiagen, 74106) according to the manufacturer’s protocol. For cDNA synthesis, 1 µg of total RNA was reverse-transcribed using the QuantiTect Reverse Transcription Kit (Qiagen, 205313) as per the manufacturer’s protocol. The resulting cDNA was used to assess MmuPV1 E4 gene expression by quantitative PCR using an Applied Biosystems ABI PRISM instrument. Primers used were as follows: MmuPV1 E1^E4 forward 5′-CAT TCG AGT CAC TGC TTC TGC-3′ and reverse 5′-GAT GCA GGT TTG TCG TTC TCC-3′; GAPDH forward 5′-GGA GAG TGT TTC CTC GTC CC-3′ and reverse 5′-ACT GTG CCG TTG AAT TTG CC-3′.

### Animals

Wild-type *FVB/N* mice (Taconic) were bred to establish the experimental cohort, and both male and female animals aged 6–8 weeks were used in MmuPV1 infection studies. The mice were housed under aseptic conditions in micro-isolator cages at the American Association for Laboratory Animal Care (AALAC)-accredited facilities at the University of Wisconsin School of Medicine and Public Health. Treatment with exogenous estrogen was performed as described previously (10, 14, 27). Briefly, male and female mice were anesthetized with 2%–5% isoflurane, and a continuous-release 17β-estradiol pellet (0.05 mg/60 days release; Innovative Research of America, Sarasota, FL, Catalog SE-121) was implanted subcutaneously into the dorsal shoulder fat pad. Estrogen treatment was initiated 5 days before or 5 days after MmuPV1 infection. *Esr1* (ERKO) mice were maintained on an *FVB/N* genetic background as described previously (28). Heterozygotes were intercrossed and offspring were genotyped by PCR to distinguish wild-type, heterozygous (*Esr1^+/−^*), and homozygous (*Esr1*^−/−^) mice, using primers that flank the targeted region; forward: 5′-CCATGGGTCATCACTGGGTC-3′ and reverse:5′ CCACAGCTTCCCTGGCATTA-3′.

### MmuPV1 isolation and infection

The cutaneous ear tissue of *FoxN1^nu/nu^* mice was mechanically scarified prior to inoculation with 2 µL of a previously prepared MmuPV1 viral stock. The resulting ear papillomas (warts) were harvested 3 months post-infection to generate concentrated crude viral preparations (29). Briefly, wart tissues were homogenized in a minimal volume of PBS and incubated at 37°C for 30 min with benzonase and Triton. The homogenates were subsequently digested with collagenase IV at 4°C for 48 h. Following centrifugation, the supernatants were treated with additional benzonase, centrifuged again, and stored in aliquots as crude virus stocks. To determine viral genome equivalents (VGE), 5–10 µL of virus stock was treated with proteinase K, run on agarose gel at serial dilutions, stained with SYBR Gold, and compared with a DNA ladder of known concentrations (NEB, N0550S) (Fig. S6). For experimental infections, quantified viral stocks with known VGE concentrations were diluted to the concentrations required for each study. Prior to inoculation, mouse ears were scarified using a 27-guage needle, and 2 µL of virus suspension (with VGE specified in the figure legends) was applied to the wounded sites. Papilloma dimensions (width, length, and height) were measured using calipers, and tumor volume was calculated as width × length × height as reported previously (30).

### MmuPV1 infection of the female reproductive tract

The cervicovaginal infections were done as reported previously (14). To synchronize mice in diestrus, animals received a subcutaneous injection of 3 mg medroxyprogesterone acetate (Amphastar Pharmaceuticals, Rancho Cucamonga, CA) 4 days before infection. On the day of inoculation, mice were anesthetized with 5% isoflurane and pretreated intravaginally with 50 µL Conceptrol gel (catalog no. 247149), containing 4% nonoxynol-9, to induce controlled epithelial disruption and enhance viral access. Four hours after Conceptrol treatment, mice were infected intravaginally with 10⁸ viral genome equivalents (VGE) of MmuPV1 delivered in 25 µL of 4% carboxymethylcellulose (Sigma; catalog no. C4888). All procedures were performed under anesthesia to minimize discomfort.

### Complete blood cell count

For each mouse, 50–100 μL of blood was collected via submandibular bleeding into ethylenediaminetetraacetic acid (EDTA)-coated tubes (BD Microtainer, Catalog 365974). Complete blood counts, including neutrophil and lymphocyte counts, were measured using a Hemavet automated hematology analyzer. When the analysis of white blood cell subpopulations was required, 10–40 μL of blood per mouse was processed for flow cytometry.

### Tissue collection for histology analysis

Wart tissues were fixed by immersing the samples in 4% paraformaldehyde (PFA) at 4°C overnight, followed by transfer to 70% ethanol. The tissues were then processed by infiltration with an increasing series of graded ethanol, xylenes, and molten paraffin. The tissues were then embedded in paraffin and sectioned into 5 µm slices. Every 10th section was stained with hematoxylin and eosin (H&E) staining. After staining, slides were dehydrated and mounted using Cytoseal XYL (Thermo Scientific, Cat. 83124). Histopathological assessment was performed by a board-certified pathologist (RH) in the Department of Pathology and Laboratory Medicine at the University of Wisconsin School of Medicine and Public Health, following a previously established scoring system for MmuPV1-related skin disease (30). Images of the stained wart sections were captured using a Zeiss AxioImager M2 microscope equipped with AxioVision software version 4.8.2 (Jena, Germany).

### Immunohistochemistry staining

For immunohistochemistry, tissue sections were first deparaffinized in xylene and rehydrated through a graded ethanol series. Heat-mediated antigen retrieval was performed using Tris-EDTA buffer (pH 9.0; 10 mM Tris base, 1 mM EDTA disodium salt, 0.05% Tween-20 in deionized water). Sections were blocked in 5% serum in phosphate-buffered saline with Tween (PBST) and then incubated with an antibody against estrogen receptor alpha (ERα), clone H-184 (Santa Cruz Biotechnology, sc-7207; 1:100 dilution). Signal development was carried out using the VECTASTAIN Elite ABC Universal Peroxidase Kit (Vector Laboratories, PK-6200), followed by detection with the DAB-Nickel Substrate Kit (Vector Laboratories, SK-4100). Slides were counterstained with hematoxylin, dehydrated, and mounted for imaging (14).

### Immunofluorescent staining

For immunofluorescence staining, formalin-fixed, paraffin-embedded (FFPE) tissue sections were deparaffinized, rehydrated, and subjected to heat-mediated antigen retrieval in Tris-EDTA buffer (pH 9.0), consisting of Tris base (10 mM), EDTA disodium salt (1 mM), and Tween-20 (0.05% per liter of DI water). The slides were blocked with 5% normal donkey serum for 1 h at room temperature, followed by overnight incubation at 4°C with purified primary antibodies. After three washes in PBST, the sections were incubated with fluorophore-conjugated secondary antibodies for 1 h at room temperature, counterstained with DAPI (1 mg/mL), and mounted using ProLong mounting medium (Invitrogen, Cat. P36984). The following antibodies were used: Ly6G (clone 1A8; BioLegend, Cat. 127601, 1:200), CD4 (clone 4SM95; eBioscience, Cat. 14-9766-82, 1:200), CD8a (clone 53-6.7; eBioscience, Cat. 16-0081-82, 1:200), F4/80 (clone BM8; eBioscience, Cat. 14-4801-82, 1:200), K14 (BioLegend, Cat. 905301, 1:1,000), anti-MmuPV1 E4 rabbit sera (a gift from Aussie Suzuki, McArdle Laboratory for Cancer Research), anti-MmuPV1 L1 rabbit sera (a gift from Christopher B. Buck, NIH), and Ki-67 (clone D3B5; Cell Signaling, Cat. 12202S, 1:200). The secondary antibodies included goat anti-rat Alexa Fluor 488, goat anti-rabbit Alexa Fluor 594, and goat anti-mouse Alexa Fluor 546 (Molecular Probes). The number of Ly6G, CD4, CD8 and F4/80 positive cells within the K14+ epithelial regions was counted for each field and averaged for each mouse wart. For quantification, 4–5 representative fields per wart were analyzed at 10× magnification, with 4–6 warts assessed per experimental group. Scale bars represent 100 μm. Positive cells were identified based on the signal detectable when compared to the no-primary antibody control. Staining in the highly keratinized layers was excluded from quantification.

### RNA *in situ* hybridization

MmuPV1 viral transcripts were detected using RNAscope 2.5 HD Assay-Brown (Advanced Cell Diagnostics, Newark, CA, USA) according to the manufacturer’s instructions with probes specific for MmuPV1 E4 (catalog no. 473281) and MmuPV1 L1 (catalog no. 473271) as described previously (30, 31). The slides were counterstained with hematoxylin before mounting and cover slipping.

### Flow cytometry

Flow cytometry was performed as previously described (30). Briefly, mouse whole blood was treated with eBioscience Red Blood Cell Lysis Buffer (catalog 00-4333-57) for 10 min at room temperature, followed by washing with PBS. The cells were then incubated with fluorophore-conjugated antibodies targeting immune cell surface markers at 4°C for 30 min. After staining, the suspensions were washed in PBS containing 5% FBS and analyzed using a ThermoFisher Attune flow cytometer. To perform flow cytometry on ear papillomas, samples were excised, trimmed of the surrounding tissue, and kept on ice in PBS. Warts were minced into ~1 mm fragments and digested in RPMI medium supplemented with the Miltenyi Mouse Tumor Dissociation Kit (Miltenyi Biotec B.V. & Co. KG, Germany, catalog no. 130-096-730), at 37°C for 30 min. The digested tissue was mechanically dissociated using a plunger of a 1 mL syringe, filtered through a 70 μm Falcon strainer (catalog 352350), and washed twice with cold PBS. Single-cell suspensions were stained with 1 μL of Live/Dead Fixable Blue dye (Invitrogen, Catalog L23105) in 500 μL PBS at 4°C for 15 min, washed with PBS containing 5% FBS, and then labeled with surface marker antibodies. Wart-derived cells were subsequently washed and fixed overnight at 4°C using an eBioscience Fixation Buffer (Catalog 00-5123-43). After fixation, the cells were washed in PBS with 5% FBS and analyzed using a ThermoFisher Attune/Cytek Aurora instrument. For compensation, eBioscience beads (catalog 01-2222-41) were stained individually with each antibody as a single-color controls. The antibody panel (anti-mouse) included: CD45 APC-Cy7 (BioLegend, clone 30-F11), CD8a FITC (Tonbo/eBioscience, clone 53-6.7), CD4 PE (Tonbo/eBioscience, clone RM4-5), Gr-1 PE-Cy5 (BioLegend, clone RB6-8C5), F4/80 BV421 (BioLegend, clone BM8), CD11b BV605 (BioLegend, clone M1/70), CD11c PE-Cy7 (BioLegend, clone N418), CD19 BV711 (BioLegend, clone 6D5), CD45R/B220 PE (BioLegend, clone RA3-6B2), Ly6G PE Cy7 (Biolegend, clone 1A8), Ly6C APC eFluor 780 (eBioscience, clone HK 1.4), CD335 (NKp46) BV 711 (Sirigen, clone 29A1.4).

### Statistics

All statistical analyses were performed using the GraphPad Prism software. For experiments involving two independent variables, a two-way ANOVA was applied. For single-variable comparisons, unpaired *t*-tests or one-way ANOVA was used as appropriate. Fisher’s exact test was used for categorical data, such as papilloma incidence (presence vs absence of lesions), while the Mann–Whitney *U* test (also called the Wilcoxon rank-sum test) was applied to non-parametric continuous data, including papilloma volume measurements. Statistical significance was determined using these tests wherever applicable. Data are presented as mean ± SEM; ns = not significant, *P < 0.05, **P < 0.005, ***P < 0.01, ****P < 0.001.

## RESULTS

### Estrogen enhances MmuPV1-induced papillomatosis in *FVB/N* mice independent of sex

**Fig 1:**
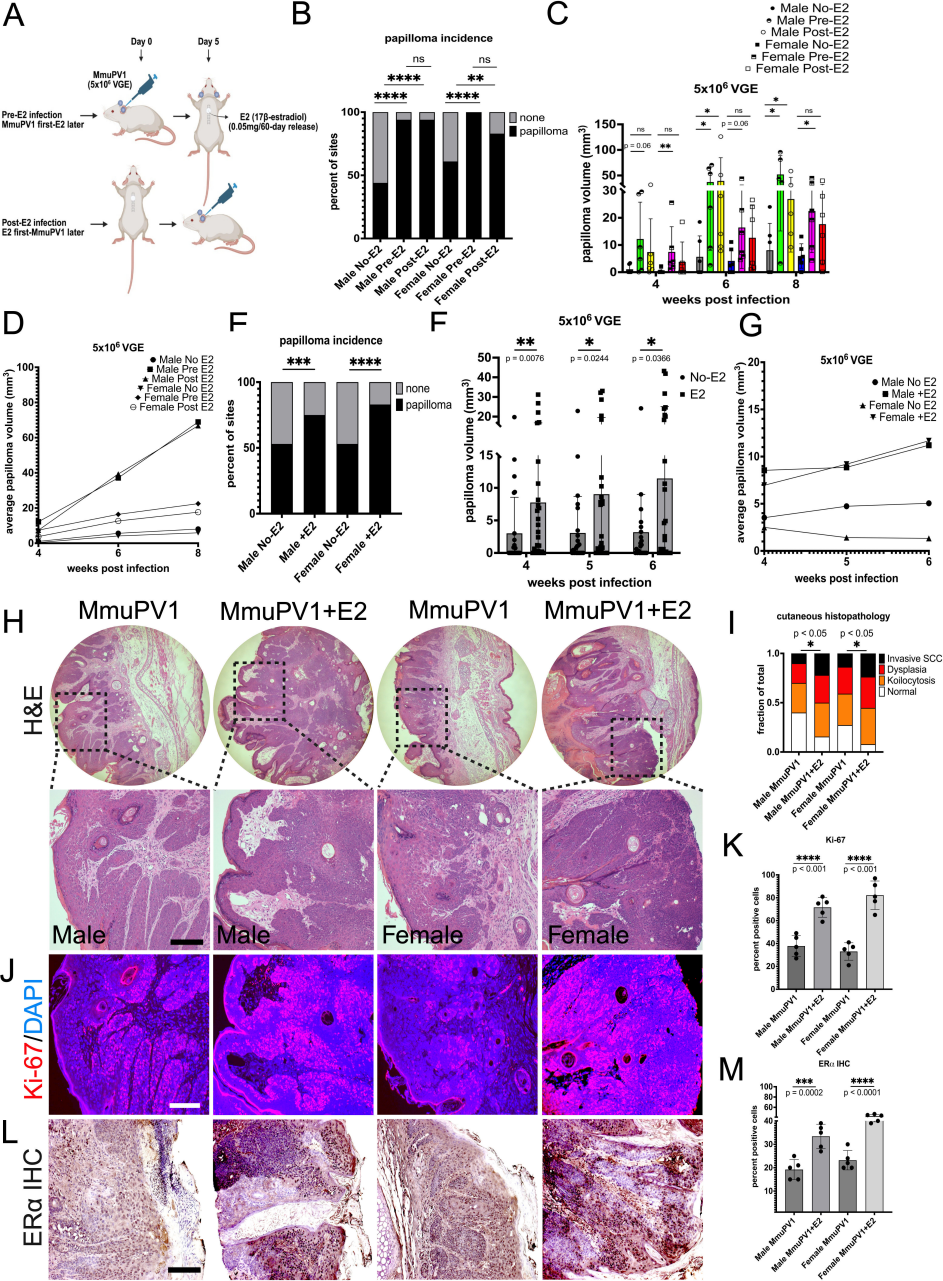
Estrogen enhances MmuPV1-induced papillomatosis in *FVB/N* mice by increasing lesion incidence and growth independent of sex. (**A**) Schematic of experimental design showing pre- and post-estrogen (E2; 17β-estradiol, 0.05 mg/60-day release) MmuPV1 infection model (illustration created in BioRender, https://BioRender.com/2btew4l). In the pre-treatment model, mice were infected with MmuPV1 and implanted with E2 pellets 5 days post-infection; in the post-treatment model, mice were implanted with E2 pellets 5 days prior to MmuPV1 infection. (**B**) Incidence of papillomatosis at 8 weeks post-infection, showing the percentage of mice developing warts. Pre- and post-E2 treatment groups exhibited similar incidence, independent of sex (Fisher’s exact test). (**C**) Wart size measured at 4, 6, and 8 weeks post-infection. Estrogen treatment significantly increased wart size in both males and females, independent of treatment timing (Mann-Whitney *U* test). (**D**) Average papilloma size increased over time in both pre- and post-E2-treated groups, with tumors consistently larger in males than females. In contrast, MmuPV1-only groups maintained very low papilloma volumes throughout 4, 6, and 8 weeks post-infection. (**E**) Incidence of papillomatosis at 6 weeks post-infection in mice pre-treated with E2 5 days post-infection. Both male and female mice showed similar incidence, indicating estrogen enhances papillomatosis independent of sex (Fisher’s exact test). (**F**) Wart size measured at 4, 5, and 6 weeks post-infection in mice pre-treated with E2 5 days post-infection. Pooled analysis of males and females revealed a significant effect of estrogen treatment, with E2-treated mice exhibiting larger wart sizes. (**G**) Average papilloma size increased in both E2-treated groups, while MmuPV1-only groups maintained very low volumes throughout 4, 5, and 6 wpi. (**H and I**) Representative 2.5 × H&E-stained images of papillomas from MmuPV1-infected mice with or without E2 treatment. Corresponding 10× zoomed in views show that E2-treated lesions display more pronounced epithelial thickening, invasive squamous cell carcinoma (SCC) features, koilocytosis, and dysplastic changes in both sexes. Histopathology scoring was performed by a board-certified pathologist on 20 sites from MmuPV1-infected males, 32 sites from MmuPV1 + E2-treated males, 22 sites from MmuPV1-infected females, and 38 sites from MmuPV1 + E2-treated females. (**J and K**) Representative Ki-67 immunostaining images showing increased proliferative activity in E2-treated papillomas compared with untreated controls in both sexes. (**L and M**) ERα immunohistochemistry demonstrates markedly higher estrogen receptor expression within papillomas from E2-treated mice in both males and females, with the strongest staining observed in females. Baseline ERα expression was low in mock-infected mice and completely absent in ERKO animals (Fig. S1C and D), confirming antibody specificity and loss of *Esr1*. Statistical significance was determined using Student’s *t*-test, Fischer’s exact test and Mann-Whitney *U* test (also known as the Wilcoxon rank-sum test), as specified. Scale bars: 100 μm. Data are presented as mean ± SEM; ns = not significant, *P < 0.05, **P < 0.005, ***P < 0.01, ****P < 0.001.

To investigate the role of estrogen in MmuPV1-induced cutaneous infection and disease, we established both pre- and post-treatment infection models in *FVB/N* mice using 17β-estradiol (E2; 0.05 mg/60-day release pellets). This concentration of E2 has previously been shown to induce persistent estrus, a well-characterized physiological state in mice that is associated with moderately elevated endogenous estrogen levels (11). In the pre-treatment model, mice were infected with MmuPV1 and implanted with E2 pellets 5 days post-infection, whereas in the post-treatment model, mice were implanted with E2 pellets 5 days prior to viral infection (Fig. 1A) (*N* = 3 for all the groups). For infection, both ears were scarified with a 27-guage needle, to facilitate viral access to the basal cells, which are the target cells of infection. Each ear was inoculated with 5 × 10^6^ viral genome equivalents (VGE) of MmuPV1, a dose sufficient to induce papillomas in ~50% of the male and female control mice. Because wound healing typically resolves within 3 weeks, papilloma incidence and size were assessed beginning at 4 weeks post-infection. Analysis of disease incidence revealed that both pre- and post-treatment regimens resulted in comparable rates of papillomatosis at 8 weeks post-infection, independent of sex (Fig. 1B). Wart growth was longitudinally assessed. Lesion size measured at 4-, 6-, and 8-weeks post-infection demonstrated that estrogen significantly enhanced wart growth in both male and female mice, regardless of treatment timing (Fig. 1C). Statistical analysis confirmed the estrogen-treated groups developed larger warts than the untreated controls. To increase the statistical power, data from males and females were pooled, revealing a consistent effect of estrogen across sexes (Fig. S1A). Analysis of the average papilloma size increased over time in both groups receiving E2, regardless of the timing of E2 administration. The tumors were consistently larger in males than in females. In contrast, the groups infected with MmuPV1 alone maintained very low papilloma volumes at 4, 6, and 8 weeks post-infection (Fig. 1D). To further validate these findings, an independent cohort of mice was examined in which the experimental group received E2 pellets implanted 5 days post-infection (*N* = 6 for each group). At 6 weeks post-infection, both male and female mice treated with E2 displayed a similar incidence of papillomatosis, reinforcing that estrogen enhances lesion penetrance independent of sex (Fig. 1E). Wart size analysis in this cohort confirmed that E2-treated mice developed significantly larger lesions than controls (Fig. 1F). Pooled analysis confirmed the growth-promoting effect of estrogen, while sex-specific analyses (Fig. S1B) indicated that the effect was consistent across sexes although it did not reach statistical significance. Analysis of the average papilloma size over time showed that tumor size increased in both E2-treated groups, with consistently larger sizes in both males and females. In contrast, groups infected only with MmuPV1 maintained very low papilloma volumes at 4, 5, and 6 wpi although the sizes were slightly higher in males than in females (Fig. 1G). The effect of estrogen on cutaneous infection and disease is consistent with earlier studies demonstrating that estrogen acts as a cofactor in papillomavirus-associated cervicovaginal disease (14, 24, 32). Histopathological analysis of lesions collected at 8 wpi revealed that E2-treated papillomas exhibited marked increases in epithelial thickness, dysplastic alterations, koilocytosis, and progression to SCC-like invasive pathology when compared to untreated MmuPV1-infected controls (Fig. 1H and I). Such rapid onset of papillomavirus-induced SCC has been previously reported (33). These findings align with previous studies showing that estrogen acts as a cofactor in papillomavirus-driven neoplasia by promoting epithelial hyperplasia and creating a permissive environment for viral persistence and progression. Immunohistochemistry demonstrated a higher percentage of Ki-67-positive cells in E2-treated lesions across sexes (Fig. 1J and K), consistent with reports that estrogen signaling synergizes with viral oncogenes to enhance epithelial proliferation (9, 11, 27). ERα immunohistochemistry revealed low baseline receptor expression in mock-treated epithelium (Fig. S1C and D), whereas ERα levels increased substantially in MmuPV1-infected papillomas and were further elevated in infected lesions supplemented with E2 in both males and females. The strongest staining was observed in E2-treated females (Fig. 1L and M), consistent with enhanced epithelial expansion and prior evidence that ERα-mediated signaling supports papillomavirus-associated epithelial proliferation and lesion maintenance (13). In contrast, no ERα immunoreactivity was detected in papillomas from ERKO mice, confirming both antibody specificity and the successful loss of *Esr1* (Fig. S1C and D). Collectively, these findings demonstrate that estrogen enhances cutaneous papilloma growth independent of sex or timing of hormone administration, in part through ERα-dependent epithelial proliferative mechanisms. These results reinforce the concept that estrogen signaling is a critical determinant of papillomavirus pathogenesis.

### Estrogen alters systemic and local immune responses by reducing circulating and tumor-infiltrating immune cells in MmuPV1-infected mice at cutaneous tissues

**Fig 2:**
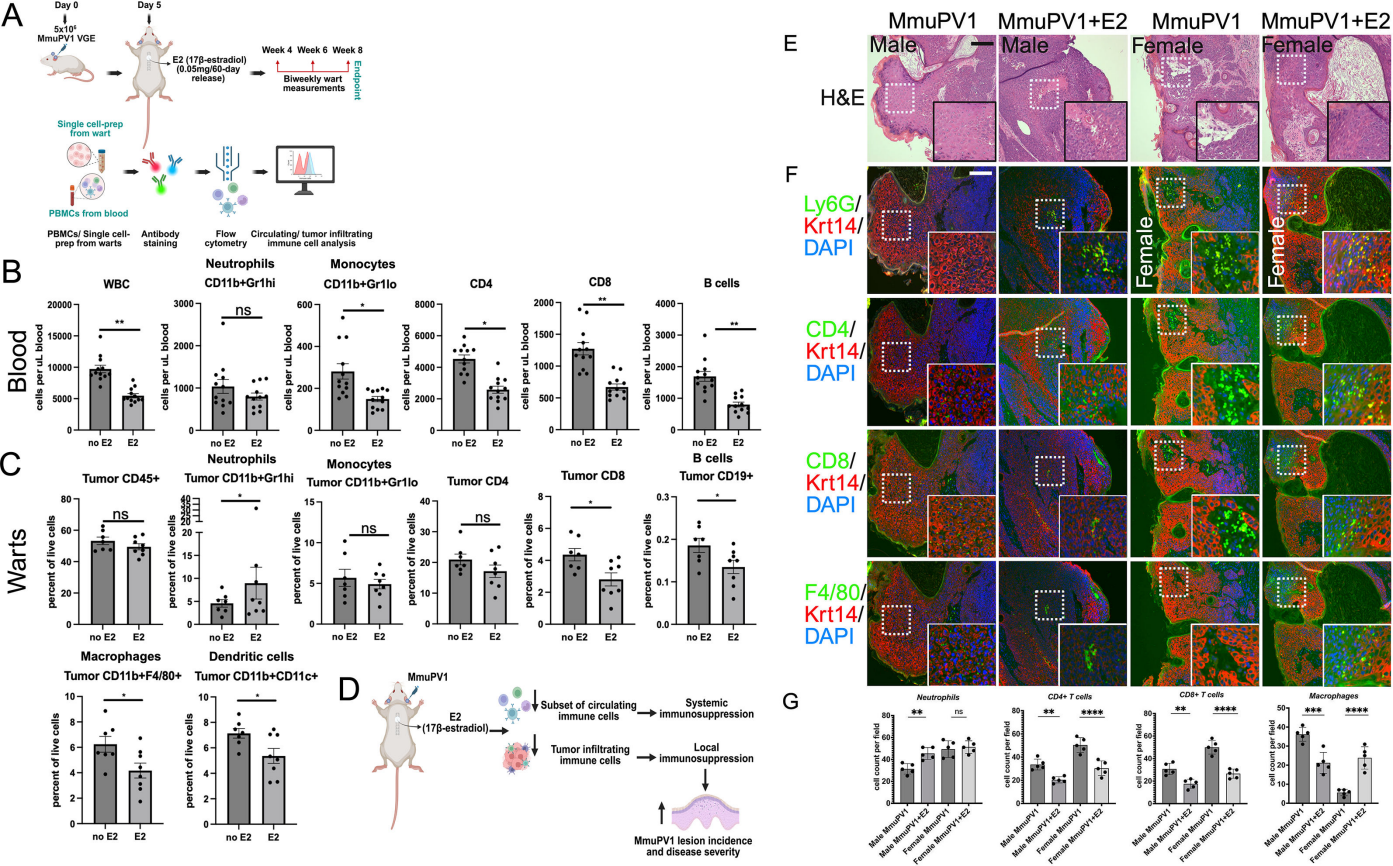
Estrogen alters systemic and local immune response by depleting circulating and tumor-infiltrating immune cells in MmuPV1-infected mice. (**A**) Experimental workflow (created in BioRender, https://BioRender.com/63w6wf8). Mice were either pre-treated or post-treated with 17β-estradiol (E2) as described in Fig. 1. For pre-treatment, mice were infected with 5 × 10^6^ MmuPV1 viral genome equivalents (VGE) on day 0 and received E2 pellets on day 5. For post-treatment, mice received E2 pellets (0.05 mg, 60-day release) on day 0 and were infected on day 5. Additional control groups received either E2 or MmuPV1 alone. Wart measurements were taken biweekly starting at week 4. Peripheral blood mononuclear cells (PBMCs) and wart tissues were collected at 8 weeks post-infection for single-cell preparation, antibody staining, and flow cytometry analysis of circulating and tumor-infiltrating immune cells. (**B and C**) Flow cytometry was performed on blood and ear wart samples collected at 8 weeks post-infection to quantify circulating and tumor-infiltrating immune cells, including CD45+, tumor CD45^+^, neutrophils (CD11b^+^Gr1 high), tumor CD11b^+^Gr1 high, monocytes (CD11b^+^Gr1 low), tumor CD11b^+^Gr1 low, CD4^+^, tumor CD4^+^, CD8^+^, tumor CD8^+^, B cells (B220^+^), tumor B cells (CD19^+^), macrophages (CD11b^+^F4/80^+^), and dendritic cells (CD11b^+^CD11c^+^). Statistical comparisons were made using unpaired Student’s *t*-tests. Data from male and female mice were pooled to increase statistical power, revealing a consistent effect of estrogen in reducing both circulating and tumor-infiltrating immune cells compared to untreated controls. Bars represent mean ± SEM. (**D**) Schematic representation of the proposed mechanism by which 17β-estradiol (E2) enhances MmuPV1-induced cutaneous disease. (Created in BioRender, https://BioRender.com/oxj4azv.) (**E**) Representative hematoxylin and eosin (H&E)-stained sections of MmuPV1-infected ear tissue from different treatment groups, showing overall tissue architecture and dysplastic changes. Dysplastic changes are more pronounced in E2-treated mice of both sexes. Scale bars = 100 µm. (**F**) Representative immunofluorescence images of wart sections stained for Ly6G (neutrophils), CD4 (T helper cells), CD8 (cytotoxic T cells), and F4/80 (macrophages), along with keratin 14 (Krt14, epithelial marker) and counterstained with DAPI (nuclei). Scale bars = 100 µm. (**G**) Quantification of immune cell infiltration per field (4 or 5 fields per wart, 4–6 warts per group). Data are presented as mean ± SEM; ns = not significant, *P < 0.05, **P < 0.005, ***P < 0.01, ****P < 0.001.

In our previous study investigating the effect of estrogen on MmuPV1 infection in the female reproductive tract, we found that estrogen alters both systemic and local immune responses during MmuPV1 infection (24). To determine whether the same is true in the context of cutaneous MmuPV1 infection, we performed flow cytometric analyses of immune cells circulating in the blood or present within papillomas in untreated or estrogen-treated MmuPV1-infected mice. Mice were either pre-treated or post-treated with 17β-estradiol (E2) pellets (0.05 mg, 60-day release) and infected with 5 × 10^6^ MmuPV1 VGE as outlined in Fig. 2A. The control groups received either E2 alone or MmuPV1 alone. Wart growth was monitored biweekly starting at 4 wpi, and immune profiling was performed at 8 wpi. Peripheral blood samples were analyzed using an automated hematology analyzer to quantify the circulating white blood cell (WBC) counts. We found that estrogen treatment reduced whole blood cell counts, as well as circulating levels of CD4+ T cells, CD8+ T cells, B cells, and monocytes, but not circulating levels of neutrophils (Fig. 2B). These differences in circulating immune cells resulting from estrogen treatment were observed in both male and female mice infected with MmuPV1. Within the ear warts of infected mice, estrogen treatment reduced the percentages of CD8+ T cells, B cells, macrophages, and dendritic cells, but increased the percentage of neutrophils (Fig. 2C). The reduction in CD4+ T cells and monocytes within the warts of estrogen-treated mice was not statistically significant. Based on these results, we propose that E2 promotes the development and progression of MmuPV1-induced cutaneous disease through immunosuppression (Fig. 2D). Our results demonstrate that estrogen reduces subsets of circulating immune cells, leading to systemic immunosuppression, and simultaneously decreases immune cell infiltration at the lesion site, causing local immunosuppression. Data from male and female mice were pooled to increase statistical power, and sex-specific analyses confirmed that estrogen-mediated immune suppression occurred independently of sex and the timing of E2 administration (Fig. S2A). Similar patterns were observed for neutrophils, CD4+ T cells, CD8+ T cells, and macrophages using immunofluorescence staining (Fig. 2E through G). E2 treatment significantly reshaped the local immune landscape, characterized by robust neutrophil infiltration in both sexes (though not statistically significant in females) and a marked increase in the presence of macrophages in males. In contrast, CD4+ and CD8+ T cell populations were dramatically reduced in E2-treated, MmuPV1-infected mice of both sexes. Immune signals were largely confined to K14-positive epithelial regions across groups, except in females infected with MmuPV1 alone, where infiltration extended into stromal areas. Collectively, these findings indicate that estrogen broadly suppresses systemic adaptive immunity while promoting localized neutrophil enrichment similar to that observed in the cervicovaginal MmuPV1 infection model (24). This dual effect of systemic suppression of adaptive immunity combined with localized neutrophil enrichment may represent a critical pathway through which hormonal factors influence papillomavirus pathogenesis and disease severity.

### Estrogen drives productive MmuPV1 infection by enhancing epithelial differentiation and viral gene expression

**Fig 3:**
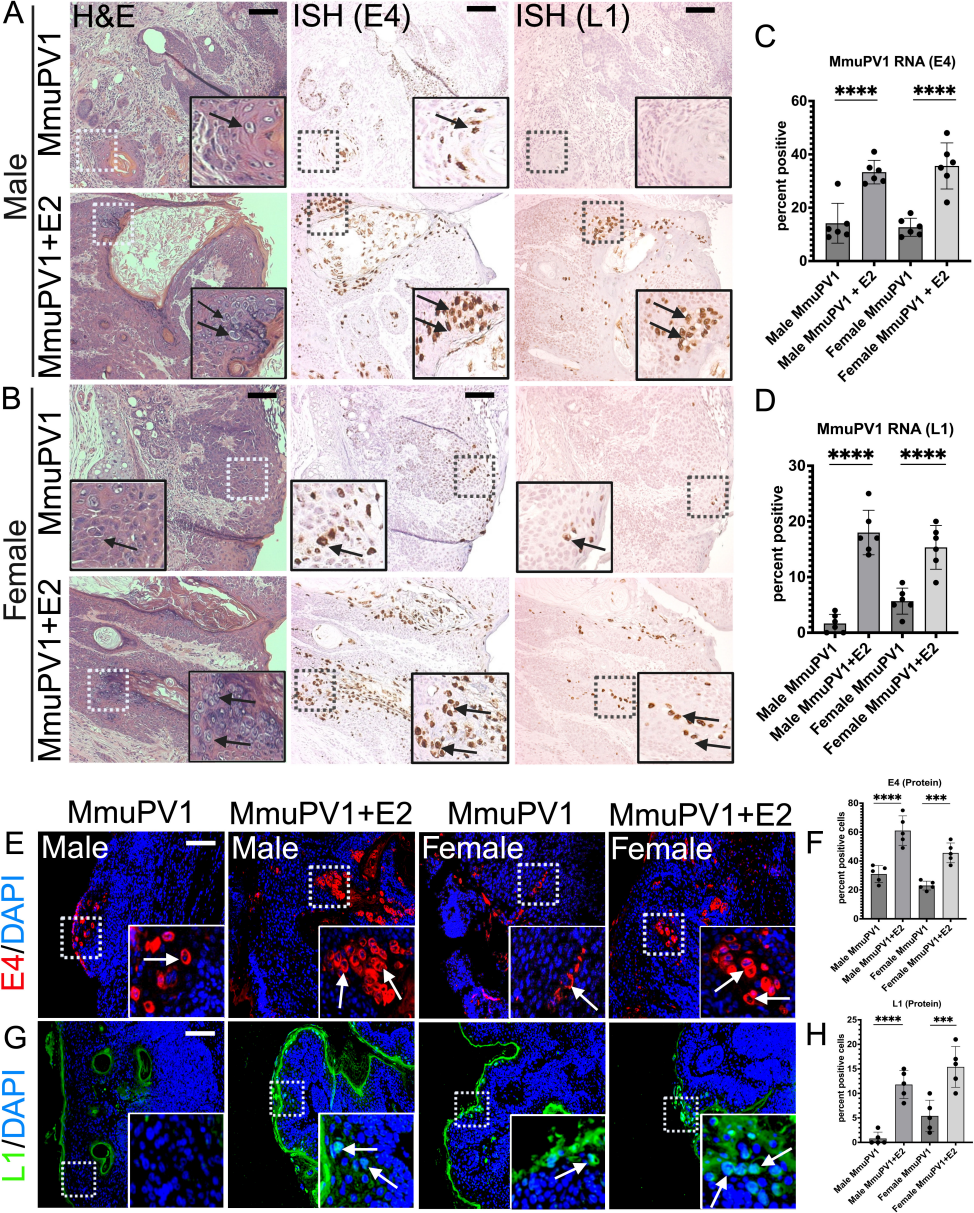
Estrogen drives productive MmuPV1 infection by enhancing epithelial differentiation and viral gene expression. (**A and B**) H&E-stained sections of MmuPV1-infected papillomas in male and female ears with or without E2 supplementation. Enlarged views highlight koilocytes (black arrows), a characteristic of papillomavirus infection. Koilocytes were more abundant in E2-treated mice compared to MmuPV1-only infected tissues. Corresponding serial sections were stained with RNAscope *in situ* hybridization (ISH) for MmuPV1 E4 and L1 RNA. Enlarged views (black arrows) show punctate brown cytoplasmic signals, indicating viral transcript localization. E4 and L1 RNA expression was significantly higher and more widespread in E2-treated tissues. The nuclei were counterstained with hematoxylin (blue/purple blue). (**C and D**) Quantification of E4 and L1 RNA-positive cells per 10× field in the male and female groups (*n* = 6 warts per group). (**E–H**) Immunofluorescence staining for E4 (red), L1 (blue), and DAPI (blue) in papillomas from male and female mice with or without E2 treatment. Enlarged views highlight the increased E4 and L1 signal intensity and distribution in E2-treated tissues. Data are presented as the mean ± SEM. Statistical significance was determined using Student’s *t*-test. Scale bars = 100 µm. ns: not significant. *P < 0.05, **P < 0.005, ***P < 0.001, ****P < 0.0001.

To determine whether estrogen influences viral gene expression and epithelial differentiation during MmuPV1-induced cutaneous papillomatosis, we analyzed papillomas from wild-type *FVB/N* MmuPV1-infected mice either untreated or treated with 17β-estradiol (E2; 0.05 mg/60-day release pellets). Eight weeks post-infection, lesions were harvested from both sexes for histopathology, RNAscope *in situ* hybridization (ISH), and immunofluorescence analysis (Fig. 3). Hematoxylin and eosin (H&E) staining revealed that E2-treated papillomas exhibited pronounced epithelial thickening and expanded spinous and granular layers compared with papillomas from untreated mice. Increased magnification images (insets) show numerous koilocytes, (cells with perinuclear halos and nuclear atypia), distributed throughout the upper epithelial strata in E2-treated lesions in both males and females, whereas koilocytes were sparse in MmuPV1-only papillomas (Fig. 3A and B). Koilocytosis is a hallmark of papillomavirus infection and reflects productive viral replication in differentiating keratinocytes, consistent with previous reports in both MmuPV1 and HPV models (10, 34, 35). To assess viral transcriptional activity, RNAscope ISH was performed using probes detecting transcripts containing the MmuPV1 E4 gene, which are present within most viral transcripts, and the L1 gene. Nuclear positivity also reflects the detection of MmuPV1 DNA genomes (31). Punctate brown signals for E4 and L1 were detected in the cytoplasm of suprabasal keratinocytes, with markedly higher density and broader distribution in E2-treated papillomas than in controls in both sexes (Fig. 3A and B). Quantitative analysis of E4 and L1 RNA-positive cells across three 10 × fields per wart (*n* = 6 warts per group) confirmed a significant increase in the viral transcript abundance in E2-treated mice (Fig. 3C and D). To determine whether these transcripts were translated, immunofluorescence staining for the E4 and L1 proteins was performed. E4 (red) and L1 (green) immunoreactivity was substantially higher in E2-treated papillomas than in untreated control papillomas in both males and females, with signals localized to the upper epithelial layers (Fig. 3E and F and 3Gto H). Together, these results suggest that hormonal signaling may broadly influence papillomavirus pathogenesis by promoting epithelial states favorable for viral replication and persistence (31, 36, 37).

### *Esr1* germline deletion prevents estrogen-mediated immunosuppression and alleviates MmuPV1-induced cutaneous disease burden

Previously, we observed that E2-induced increases in wart size and incidence, together with reductions in circulating immune cells, are mediated by estrogen receptor alpha (ERα) in the context of cervicovaginal infection (6, 13). To determine whether these ERα-dependent effects extend to cutaneous MmuPV1 disease, we utilized a genetically engineered strain of mice with germline knockout of the *Esr1* gene (ERKO), which encodes for the ERα receptor (28) backcrossed onto the *FVB/N* background. The cutaneous epithelium of the ears of male and female ERKO mice and WT controls were infected with 10^8^ VGE of MmuPV1. Both the WT (*n* = 3) and ERKO cohorts (*n* = 2 for males, *n* = 4 for females) were supplemented with E2, thereby isolating the requirement for ERα by holding hormone exposure constant across genotypes. This study aimed to test whether the potentiating effects of estrogen on enhanced wart penetrance/growth and systemic immune suppression require ERα signaling.

**Fig 4:**
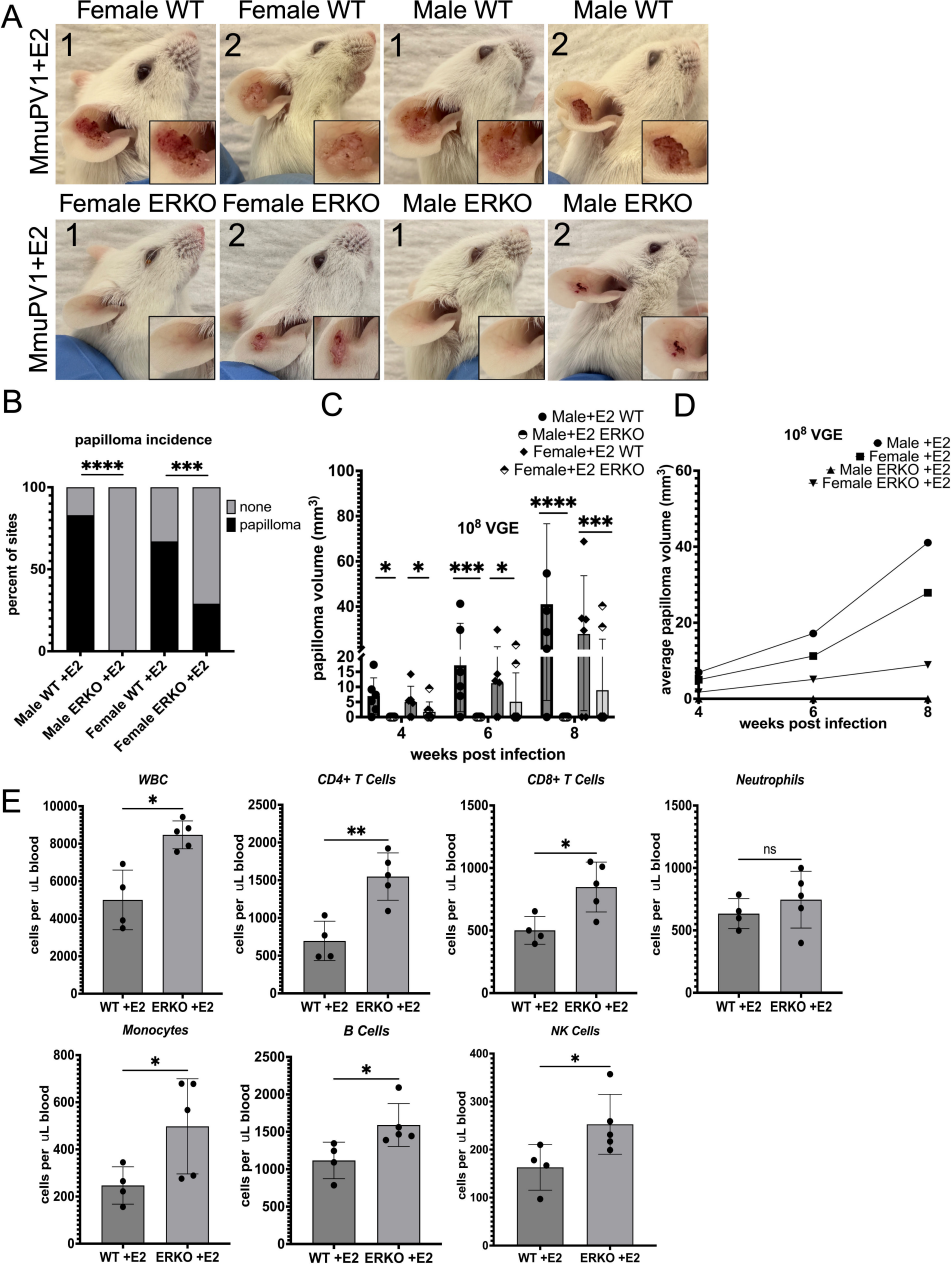
*Esr1* germline deletion prevents estrogen-mediated immunosuppression and alleviates MmuPV1-induced cutaneous disease burden. (**A**) Representative images of papilloma formation at infection sites of WT and ERKO mice treated with E2. (**B**) Quantification of papilloma incidence demonstrates a significant reduction in ERKO mice compared to WT controls, with ERKO animals showing fewer sites developing papillomas. (**C**) Papilloma volume measurements over time (4, 6, and 8 weeks post-infection) in estrogen-treated, MmuPV1-infected WT and ERKO mice. (**D**) Papilloma volumes increased in both male and female E2-treated mice, while ERKO mice showed consistently minimal lesion growth across 4–8 wpi. (**E**) Flow cytometry analysis of circulating immune cell populations (total white blood cells [WBC], CD4^+^ and CD8^+^ T cells, B cells, neutrophils, monocytes, and NK cells) in estrogen-treated, MmuPV1-infected WT and ERKO mice. Data are presented as mean ± SEM. Statistical significance was determined using Student’s *t*-test, Fischer’s exact test and Mann–Whitney *U* test (also called the Wilcoxon rank-sum test), as specified. ns: not significant. *P < 0.05, **P < 0.005, ***P < 0.001, ****P < 0.0001.

As illustrated in representative images of papilloma formation on infected ears (Fig. 4A), E2-treated WT mice developed large and prominent papillomas, whereas E2-treated ERKO mice exhibited markedly reduced papilloma formation, indicating that ERα is required for E2-driven papilloma growth. Quantification of papilloma incidence demonstrated a significant reduction in ERKO mice compared to WT controls, with infected ERKO animals showing significantly less papilloma development at the infected sites (Fig. 4B). Longitudinal measurements of papilloma volume at 4, 6, and 8 weeks post-infection showed that E2-treated WT mice exhibited progressive lesion enlargement in both male and female mice, whereas E2-treated ERKO mice maintained significantly smaller papillomas throughout the study period, regardless of sex, confirming that ERα is required for estrogen-driven papilloma growth (Fig. 4C). Analysis of average papilloma size over time revealed that tumor volumes increased in both male and female E2-treated groups, whereas ERKO mice consistently exhibited negligible to very low papilloma sizes at 4, 6, and 8 weeks post-infection (wpi), regardless of sex (Fig. 4D).

We performed flow cytometric analysis of circulating immune cell populations in MmuPV1-infected, E2-treated WT, and ERKO mice (data pooled from male and female mice for statistical comparison because of limited male ERKO numbers; sex-specific trends were consistent). E2 treatment in WT mice was associated with persistently low levels of total white blood cells (WBCs), CD4+ and CD8+ T cells, B cells, and NK cells, consistent with the E2-induced immunosuppression previously reported in cervicovaginal disease models and the cutaneous experiments described above in Fig. 2 (24). In contrast, E2-treated ERKO mice showed significantly higher levels of these immune cells, suggesting that *ERα* mediates the immunomodulatory effects of E2 (Fig. 4E). Notably, neutrophil levels did not differ significantly between WT and ERKO mice. These findings are consistent with and extend previous studies demonstrating the essential role of ERα in HPV-associated cervical disease and HNSCC carcinogenesis (38–41). Together, these data support and strengthen the concept that estrogen signaling is critical for papillomavirus pathogenesis and disease development, likely mediating these effects through immunomodulatory mechanisms during MmuPV1 infection, aligning with prior work on ERα knockout models in HPV pathogenesis.

### *Esr1* deletion impairs MmuPV1 establishment and reduces viral gene transcript levels

**Fig 5:**
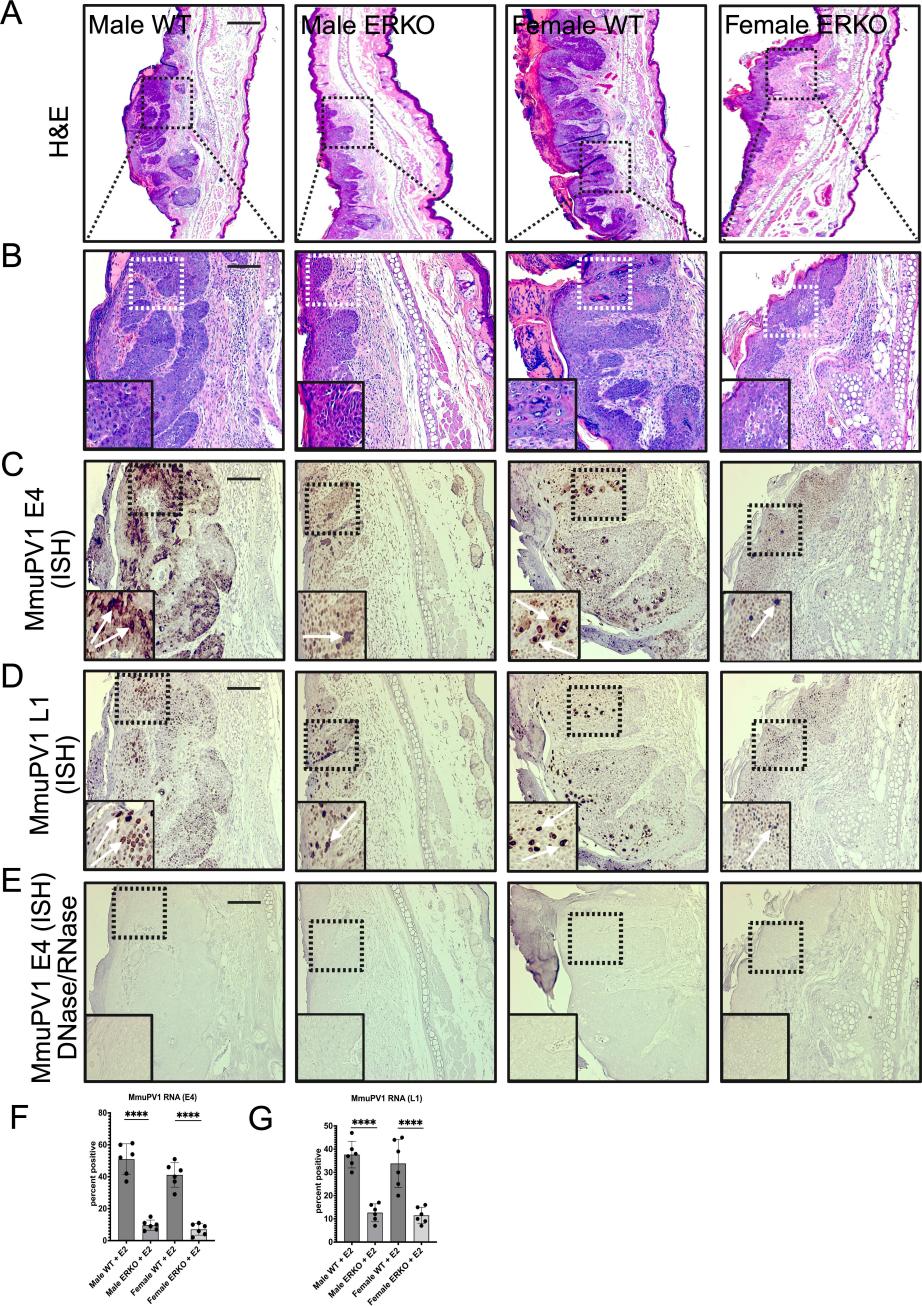
*Esr1* deletion impairs MmuPV1 establishment and reduces viral gene transcript levels. (**A and B**) Histopathological analysis of ear lesions from male and female wild-type (WT) and Esr1-knockout (ERKO) mice infected with 10⁸ MmuPV1 VGE in the presence of estrogen. WT mice of both sexes developed extensive epithelial hyperplasia, dysplasia, koilocytosis, and focal regions resembling invasive squamous cell carcinoma, confirming robust MmuPV1 disease establishment. Insets show magnified views of representative pathological features. In contrast, ERKO mice exhibited markedly attenuated epithelial changes, with minimal hyperplasia and reduced dysplasia. (**C and D**) RNAscope detection of MmuPV1 E4 and L1 transcripts in WT and ERKO lesions. WT tissues showed abundant and widespread E4 and L1 signal, whereas ERKO lesions contained substantially reduced transcript levels, indicating impaired viral gene expression in the absence of *Esr1*. (**E**) DNase/RNase-treated control sections showing complete loss of E4 staining, confirming RNAscope assay specificity. (**F and G**) Quantification of RNAscope puncta for E4 and L1 transcripts demonstrating significantly decreased viral RNA abundance in ERKO tissues compared to WT controls. Data are presented as mean ± SEM. Statistical significance was determined using Student’s *t*-test, Fischer’s exact test and Mann–Whitney *U* test (also called the Wilcoxon rank-sum test), as specified. Scale bars = 100 µm. ns: not significant. *P < 0.05, **P < 0.005, ***P < 0.001, ****P < 0.0001.

To investigate the impact of *Esr1* deletion on MmuPV1-induced cutaneous disease, we analyzed ear lesions from mice infected with 10⁸ MmuPV1 VGE in the presence of estrogen. As shown in Fig. 5A, both male and female wild-type (WT) mice developed extensive epithelial hyperplasia, dysplasia, koilocytosis, and focal areas resembling invasive squamous cell carcinoma, confirming efficient MmuPV1 disease establishment, pathological features highlighted in the magnified views (Fig. 5B). In contrast, *Esr1*-knockout (ERKO) mice of both sexes exhibited markedly attenuated epithelial changes, with minimal hyperplasia and reduced dysplastic architecture, indicating a strong dependence on *Esr1* signaling for lesion progression. Consistent with the histopathology findings, RNAscope analyses showed abundant E4 and L1 viral RNA transcripts in WT lesions, whereas ERKO lesions displayed substantially reduced signal intensity and distribution (Fig. 5C and D), reflecting impaired viral transcriptional activity in the absence of *Esr1*. Importantly, DNase/RNase-treated controls showed complete loss of E4 and L1 staining (Fig. 5E), confirming the specificity of the RNAscope assay. Quantitative analysis of RNAscope puncta (Fig. 5F and G) demonstrates significant reductions in viral RNA abundance in ERKO tissues. Together, these findings establish that *Esr1* is critical for robust MmuPV1 infection, viral gene expression, and the development and severity of cutaneous disease. To determine whether estradiol-mediated increases in viral signal derive from viral RNA or viral DNA, adjacent sections were pre-treated with DNase (Fig. 5A and C) or RNase (Fig. 5B and D) prior to probe hybridization. Estradiol-treated WT lesions retained strong E4 and L1 puncta following RNase digestion, whereas signal was abolished by DNase treatment, except for DNase-treated WT females demonstrating that the majority of detectable RNAscope signal reflects viral DNA rather than RNA transcripts (Fig. S5A through D). Quantification of E4 (Fig. 5E) and L1 (Fig. 5F) signals demonstrates significantly higher viral DNA signal in WT mice compared with ERKO mice, confirming that *Esr1*-mediated estrogen signaling is required for robust MmuPV1 replication.

## DISCUSSION

Papillomaviruses are highly adapted pathogens that exploit epithelial differentiation and host immune modulation to establish persistent infections and, in some cases, drive neoplastic progression (42). Although the role of estrogen as a cofactor in HPV-associated cervical and oropharyngeal cancer has been investigated, its impact on cutaneous papillomavirus pathogenesis has remained largely unexplored. Understanding how hormonal cues intersect with viral life cycles is critical because hormone-driven epithelial remodeling and immune suppression play major roles in clinically relevant disease progression. Our findings demonstrate that estrogen is a potent cofactor for MmuPV1 pathogenesis, increasing wart penetrance and accelerating lesion growth in *FVB/N* mice independent of sex and of the timing of hormone administration. Estrogen exposure was associated with enhanced cutaneous epithelial proliferation, heightened histopathologic cutaneous disease severity (dysplasia, koilocytosis, and features of invasive SCC), suppression of systemic and local adaptive immunity with a relative enrichment of neutrophils, and amplification of viral gene expression (E4/L1 RNA and protein) within differentiating suprabasal keratinocytes. Together, these data support a model in which estrogen-mediated signaling creates a permissive epithelial and immunological niche that facilitates productive papillomavirus infection and disease progression.

Decades of work using HPV transgenic mouse models have established estrogen as both necessary and sufficient for the development and maintenance of cervical neoplasia driven by viral oncogenes, with tumor regression upon estrogen withdrawal (6, 10, 24, 38, 43–45). Our observations in a cutaneous papillomavirus model parallel these seminal reports: E2 consistently increased lesion incidence and size across the sexes (Fig. 1B through G; Fig. S1A and B), enhanced epithelial hyperplasia, dysplastic changes, koilocytosis, and progression to SCC-like invasive pathology (Fig. 1H and I), augmented Ki-67 positivity (Fig. 1J and K), and upregulation of ERα within both male and female cutaneous papillomas (Fig. 1L and M; Fig. S1C and D), indicating a common mechanistic axis in which estrogen synergizes with viral factors to drive epithelial proliferation and transformation (10, 11). Although these cutaneous lesions exhibit histologic features consistent with squamous cell carcinoma, the rapid onset of SCC-like pathology in mice reflects the accelerated kinetics of experimental papillomavirus models and does not replicate the prolonged, multistep carcinogenic trajectory characteristic of human HPV-associated squamous carcinogenesis. In humans, viral oncogene expression must be accompanied by the gradual accumulation of additional genetic and epigenetic alterations over many years; thus, the mouse model captures the cooperative interaction between estrogen and viral factors but not the temporal or evolutionary complexity of human disease. While the mouse system allows us to study how estrogen modulates viral gene expression, epithelial proliferation, and immune responses, it does not model the long-term persistence, clonal selection, and progressive genomic instability that underpin human papillomavirus-driven carcinogenesis. Instead, the model reflects an accelerated, SCC-like outcome that is useful for dissecting mechanistic interactions between host hormonal signaling and viral oncogenes but should not be interpreted as a direct temporal analog of human cancer progression.

Notably, the sex-independence of the estrogen effect suggests that circulating hormone levels and ERα signaling, rather than sex-specific factors, dominate cutaneous papillomavirus disease. This aligns with clinical and translational observations that the hormonal milieu, including exogenous estrogen, can modulate papillomavirus-associated disease risk and trajectory, particularly in tissues containing metaplastic or transformation-prone epithelia (46). By showing equivalent levels of enhancement of wart growth in male and female mice with E2 supplementation, our study extends the role of estrogen as a host cofactor beyond the cervix to the skin, underscoring the systemic impact of estrogen-dependent signaling on papillomavirus biology.

The E2-induced immune remodeling in the skin was striking. The immunomodulatory effects of estrogen are complex and often suppress adaptive immunity while reshaping the innate responses (47, 48). Estrogen profoundly altered systemic and local immunity during MmuPV1 cutaneous infection (Fig. 2B through G). Our study demonstrated that E2-treated mice exhibited reduced circulating leukocytes and a broad depletion of adaptive immune subsets (CD4+ and CD8+ T cells, B cells, monocytes, macrophages, and dendritic cells), both systemically and within warts. In contrast, Ly6G^+^ neutrophils were enriched and most pronounced in the tumor microenvironment of E2-treated lesions, despite lower systemic counts, suggesting localized inflammatory recruitment. Notably, our previous work demonstrated the induction of neutrophil chemoattractants in the stroma of HPV transgenic mice even in the absence of estrogen; however, estrogen markedly amplified their expression within the stromal compartment (9). Within lesions, CD45 + immune cells were markedly reduced, due, at least in part, to a reduction in macrophages (CD11b^+^F4/80^+^) and dendritic cells (CD11b^+^CD11c^+^) (Fig. 2B and C), indicating impaired innate immune infiltration. Importantly, sex-specific analyses verified that these immunological changes occurred independently of sex and timing of E2 administration (Fig. 2A). Immunofluorescence analysis confirmed significant reductions in CD4+ and CD8+ T cells across sexes, while macrophage depletion was significant in males only (Fig. 2F and G). Collectively, these findings indicate that estrogen promotes an immunosuppressive microenvironment, reducing adaptive surveillance while favoring conditions that support viral persistence and epithelial proliferation, which is consistent with prior HPV paradigms (24). Our findings further indicate that estradiol’s effects on lesion progression and viral productivity arise not only from epithelial-intrinsic mechanisms but also from substantial reshaping of the local immune microenvironment. Prior work from our lab (49) and current data support that immune pressure is a major regulator of papillomavirus lesion growth, and estradiol modulates key immune cell populations in ways that can enhance viral persistence. Future studies using defined immune modulators will be essential to disentangle estradiol-driven epithelial proliferation from its systemic and local immunoregulatory effects.

To explore the basis of estrogen-mediated immune suppression during MmuPV1 cervicovaginal infection, we examined whether the observed reductions in circulating immune cells could be attributed to altered hematopoiesis or sequestration within the bone marrow. However, multiparameter flow-cytometric profiling of major leukocyte subsets across treatment groups did not reveal any E2-dependent changes in bone marrow immune composition, indicating that this compartment is unlikely to account for the systemic immune suppression observed (Fig. S3B). These findings suggest that estrogen acts through alternative, tissue-specific mechanisms that remain to be defined. Future studies employing conditional, tissue-specific *Esr1* knockout models targeting epithelial, stromal, endothelial, or specific immune cell populations will be essential for identifying the precise cellular pathways through which estrogen modulates antiviral immunity.

H&E staining revealed that E2-treated mice developed a markedly thickened epidermis with pronounced epithelial hyperplasia and expanded spinous and granular layers compared to MmuPV1-only controls (Fig. 2E and 3A and B). Estrogen-treated lesions exhibited architectural distortion with papillomatous projections, increased keratinization, and dysplastic features, along with increased koilocytosis, which is a hallmark of productive papillomavirus infection. These changes were consistent across both sexes, indicating that estrogen-driven epithelial remodeling occurs independently of sex and likely contributes to enhanced viral persistence and progression. Papillomaviruses couple their life cycle to host keratinocyte differentiation; late gene expression and virion assembly occur in the suprabasal layers where cells exit the cell cycle. RNAscope and immunofluorescence showed markedly higher and broader E4/L1 RNA/protein signals in E2-treated lesions (Fig. 3A through H), consistent with enhanced late-phase viral activity (2, 50). Notably, diminished CD8+ T cell infiltration, which is critical for the control of papillomavirus infection, may directly impair immune surveillance and permit productive viral replication (51–53), as evidenced by increased E4 expression with estrogen treatment. Furthermore, the concurrent elevation of L1 protein alongside E4 indicates that E2 not only promotes viral genome amplification and late gene expression but also facilitates capsid protein synthesis, a key step in productive infection. Prior mechanistic work suggests that estrogen can promote epithelial hyperplasia and the persistence of viral genomes by augmenting cell cycle entry and altering differentiation programs (54). Our cutaneous data provide direct histological and molecular evidence for these concepts in MmuPV1 infection. To determine whether the estrogen-mediated increase in viral gene expression reflects a direct effect, we performed *in vitro* assays using primary keratinocytes transfected with re-circularized MmuPV1 genomes. Estradiol treatment significantly increased early viral transcripts in these cells compared to DMSO control, while no viral transcripts were detected in estradiol-treated wild-type keratinocytes (Fig. S4B). These data support a direct transcriptional influence of estrogen on the viral genome. However, we agree that additional studies are needed to fully define the mechanism, including whether estrogen alters transcription factor occupancy or chromatin accessibility at the viral promoter.

Our findings align well with earlier reports that immune evasion and local immunosuppression are central to the progression from benign lesions to high-grade neoplasia in HPV-associated disease (55, 56), and they suggest that hormonal cues can tip this balance in the skin similar to the cervicovaginal tract. Our extended analysis of *Esr1* germline knockout mice provides direct genetic evidence for this model. Strikingly, MmuPV1-infected ERKO mice exhibited markedly reduced papilloma incidence and size compared with WT controls (Fig. 4A through C), revealing that ERα is essential for the E2-mediated enhancement of the cutaneous papillomavirus disease burden. Tumor growth analysis showed that papilloma volumes increased progressively in both male and female E2-treated mice, whereas ERKO animals exhibited consistently minimal lesion development across all time points (4–8 wpi), irrespective of sex (Fig. 4D). This genetic ablation not only limited lesion growth but also prevented the systemic immunosuppressive effects of E2, on circulating WBC, CD4+ and CD8+ T cells, monocytes, B cells, and NK cells, with neutrophils showing a partial but non-significant recovery (Fig. 4E). These findings indicate that ERα signaling is a critical upstream regulator of both epithelial and immune mediators of papillomavirus pathogenesis. Importantly, these results align with and extend prior work in HPV transgenic models demonstrating that ERα is required for estrogen-driven cervical and head and neck carcinogenesis and synergizes with viral oncogenes to promote neoplastic progression (31, 32, 35, 36). Our study demonstrates that this paradigm is not limited to the cervicovaginal tract but also extended to cutaneous papillomavirus infections, reinforcing the concept that ERα-dependent transcriptional programs generate a permissive epithelial niche that supports viral persistence and disease progression. Conversely, genetic deletion of ERα mitigates these effects, reducing the disease burden and restoring immune competence. Histopathological examination of ear lesions showed that WT mice developed extensive hyperplasia, dysplasia, koilocytosis, and SCC-like changes, whereas ERKO mice exhibited markedly attenuated epithelial alterations with minimal dysplasia (Fig. 5A and B). RNAscope analyses demonstrated abundant E4 and L1 viral transcripts in lesions of both male and female WT mice, while ERKO tissues contained significantly reduced signals (Fig. 5C and D), indicating that ERα is necessary for robust viral gene expression. Control tissues digested with DNase/RNase lacked detectable signal (Fig. 5E), confirming assay specificity. Quantification of viral RNA puncta substantiated the marked transcriptional deficit in ERKO mice (Fig. 5F and G). These findings indicate that ERα not only modulates epithelial susceptibility and immune tone but also directly influences the viral transcriptional program, likely through effects on molecular states required for late-stage papillomavirus gene expression. Our nuclease-digestion analysis further clarified the origin of the RNAscope signal. Estradiol-treated lesions retained strong E4 and L1 puncta after RNase digestion but lost signal following DNase treatment, indicating that the estradiol-dependent increase reflects viral DNA rather than RNA transcripts. This pattern suggests that estradiol enhances viral genome amplification and overall viral turnover, consistent with productive infection (Fig. S5A through F).

In high-risk human HPV infections, estrogen is also known to exert direct regulatory effects on the viral life cycle that extend beyond immune modulation. ERα can bind to estrogen-responsive elements within the HPV long control region (LCR), enhancing promoter activity and increasing transcription of the E6 and E7 oncogenes, which are central drivers of viral persistence and neoplastic progression (38, 57). Estrogen has additionally been reported to influence the stability and turnover of viral oncoproteins (6, 11), further amplifying their functional impact. These well-established mechanisms in human HPV biology provide an important translational context for our findings, suggesting that the estrogen-dependent enhancement of MmuPV1 E1^E4 and L1 expression may reflect conserved hormone-responsive pathways across papillomaviruses. Highlighting these parallels strengthens the relevance of our study and supports the broader concept that modulation of estrogen receptor signaling may hold therapeutic value in HPV-associated disease. It is important to note that the immune microenvironment varies substantially across anatomical sites where HPV causes disease in humans (42). Mucosal tissues such as the cervix and the tonsillar crypts contain specialized lymphoid structures and distinct antigen-presenting cell populations that shape local antiviral immunity in ways that differ from cutaneous epithelia (58–61). These site-specific immune features are particularly relevant in settings such as the cervix of individuals on ART or the lymphoid-rich oropharynx, where local immune surveillance plays a critical role in HPV persistence and neoplastic progression (62, 63). While our study focuses on cutaneous infection, the estrogen-dependent modulation of local immunity observed here may intersect with these unique mucosal immune landscapes, underscoring the need to consider tissue-specific immunity when translating findings to human disease.

**Fig 6:**
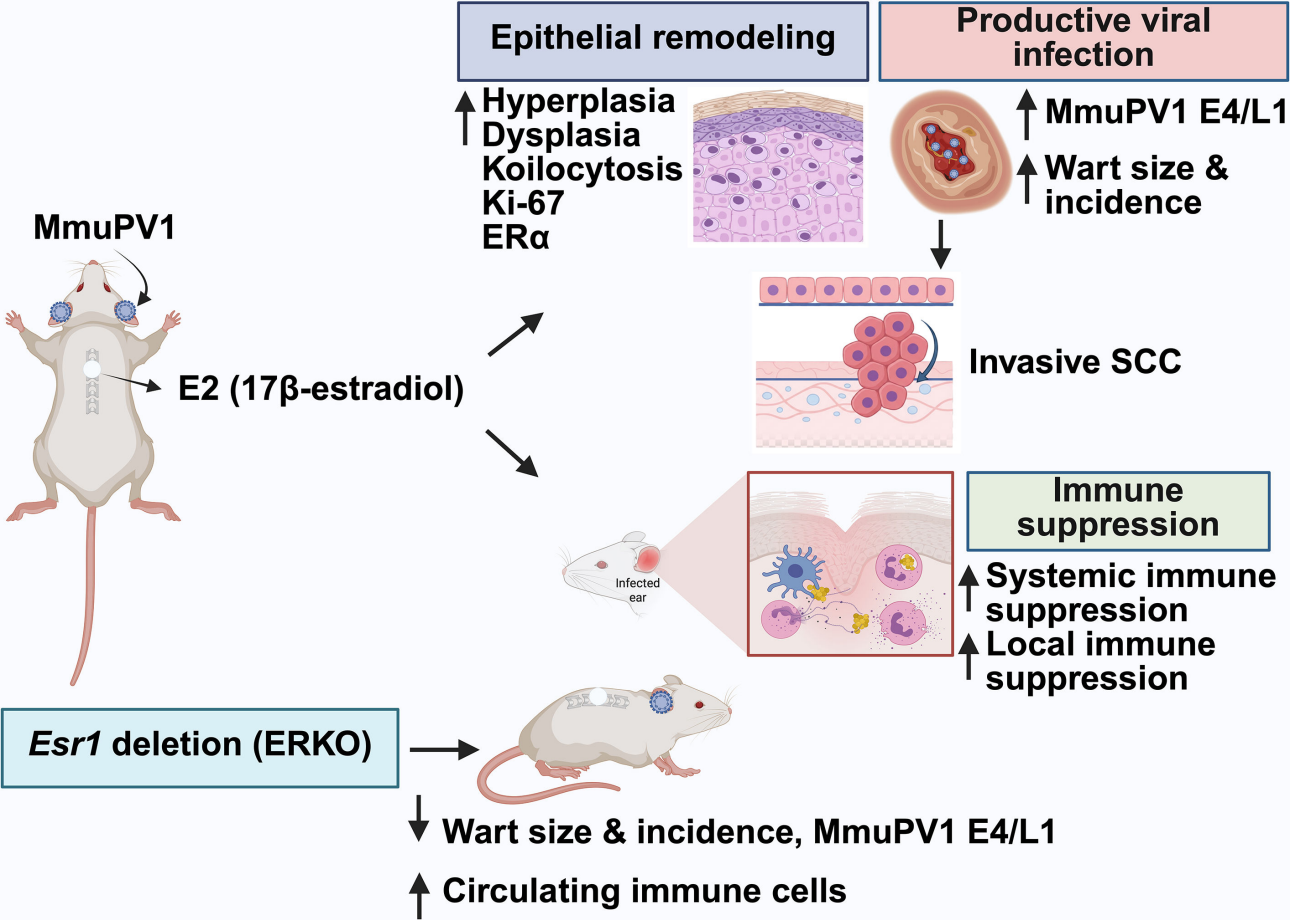
Estrogen-*Esr1* axis controls cutaneous papillomavirus disease incidence and severity. In MmuPV1-infected mice, E2 (17β-estradiol) treatment induces marked epithelial changes including hyperplasia, dysplasia, koilocytosis, elevated Ki-67, and heightened ERα activity creating a permissive environment for viral persistence. Estrogen further amplifies productive infection, shown by increased E4 and L1 RNA and protein expression, leading to larger papillomas, higher lesion incidence, and progression toward invasive SCC-like pathology. E2 also dampens both systemic and local immunity, reducing circulating CD4^+^ and CD8^+^ T cells, B cells, NK cells, and monocytes, while enriching neutrophils within lesions collectively weakening antiviral surveillance. In contrast, *Esr1* knockout (ERKO) mice show markedly reduced papilloma incidence and size, decreased MmuPV1 E4 and L1 transcript levels, and restoration of immune cell populations, highlighting ERα as a central mediator of estrogen-driven epithelial, immunologic, and viral responses during cutaneous MmuPV1 infection. Schematic diagram created in BioRender (U. Sheikh, 2026, https://BioRender.com/l9l5mb0).

Together, our findings identify ERα as a potential therapeutic target for hormone-responsive papillomavirus-associated diseases and support a unified model in which ERα acts as a central regulator linking hormonal signaling, epithelial proliferation, and immune modulation (Fig. 6). This genetic evidence strengthens the translational rationale for ERα-targeted interventions such as selective estrogen receptor modulators or antagonists as potential strategies to limit papillomavirus persistence and progression across both mucosal and cutaneous epithelia.

### Conclusion

Estrogen promotes cutaneous papillomavirus disease by suppressing adaptive immune surveillance, thereby creating a permissive niche for productive infection and lesion progression. Our data extend the established role of estrogen as a cofactor in HPV-associated cervicovaginal neoplasia to MmuPV1-induced cutaneous warts, demonstrating that these effects occur independently of sex and the timing of hormone administration. Importantly, genetic ablation of *Esr1* abrogated E2-driven papilloma growth and suppression of immune cell populations, and markedly reduced MmuPV1 viral gene transcription, confirming that ERα is indispensable for estrogen-mediated epithelial remodeling and immunosuppression. These findings align with those of previous studies in mucosal HPV models and establish ERα as a central node linking hormonal signaling, epithelial proliferation, and immune modulation in papillomavirus pathogenesis. Targeting the ERα-epithelium-immunity axis offers a rational therapeutic strategy to reduce viral persistence and disease burden across mucosal and cutaneous sites, with implications for hormone-related risk modulation and intervention in papillomavirus-associated diseases.

## Data Availability

All study data are included in the article and/or in the supplemental material.

## SUPPLEMENTAL MATERIAL

Supplemental Material mbio.00783-26-s0001.docx Fig. S1-S6.

ASM does not own the copyrights to Supplemental Material that may be linked to, or accessed through, an article. The authors have granted ASM a non-exclusive, world-wide license to publish the Supplemental Material files. Please contact the corresponding author directly for reuse.
