# Supplementary material for: Estrogen receptor α (Esr1) mediates estrogen’s ability to promote papillomavirus-induced cutaneous disease in male and female mice
Source: mBio. 2026 May 29;17(7):e00783-26. doi: 10.1128/mbio.00783-26 (PMC13343891; doi:10.1128/mbio.00783-26)
Supplement: Supplemental Material — Fig. S1-S6. [file mbio.00783-26-s0001.docx]

**Supplementary information for:**

**Estrogen Receptor α (*Esr1*) Mediates Estrogen’s Ability to Promote Papillomavirus-induced Cutaneous Disease in Male and Female Mice**

Sheikh A. Umar^1^, Wei Wang^1,2^*, Donghwan Jeon^1^, Lam Khue Pham^1^, Ella T. Ward-Shaw^1^, Denis Lee^1^, Rong Hu^3,4^, Andrea Bilger^1^, Megan E. Spurgeon^1,4,5^*, Paul F. Lambert^1,4^*

^1^McArdle Laboratory for Cancer Research, Department of Oncology, University of Wisconsin School of Medicine and Public Health, Madison, Wisconsin, United States

^2^Department of Microbiology, Genetics, & Immunology, Michigan State University, East Lansing, Michigan, United States

^3^Department of Pathology and Laboratory Medicine, University of Wisconsin School of Medicine and Public Health, Madison, Wisconsin, United States

^4^University of Wisconsin Carbone Cancer Center, University of Wisconsin School of Medicine and Public Health, Madison, Wisconsin, United States

^5^John W. and Jeanne M. Rowe Center for Research in Virology, Morgridge Institute for Research, Madison, Wisconsin, United States

***Correspondence:** PFL [plambert@wisc.edu](mailto:plambert@wisc.edu;); MES [megan.spurgeon@wisc.edu](mailto:megan.spurgeon@wisc.edu)

WW [wangw151@msu.edu](mailto:wangw151@msu.edu)

**Running title:** *Esr1* Drives Estrogen-Dependent MmuPV1 Papillomatosis

**Supplement Information:** Supplementary Figures S1-S6

**
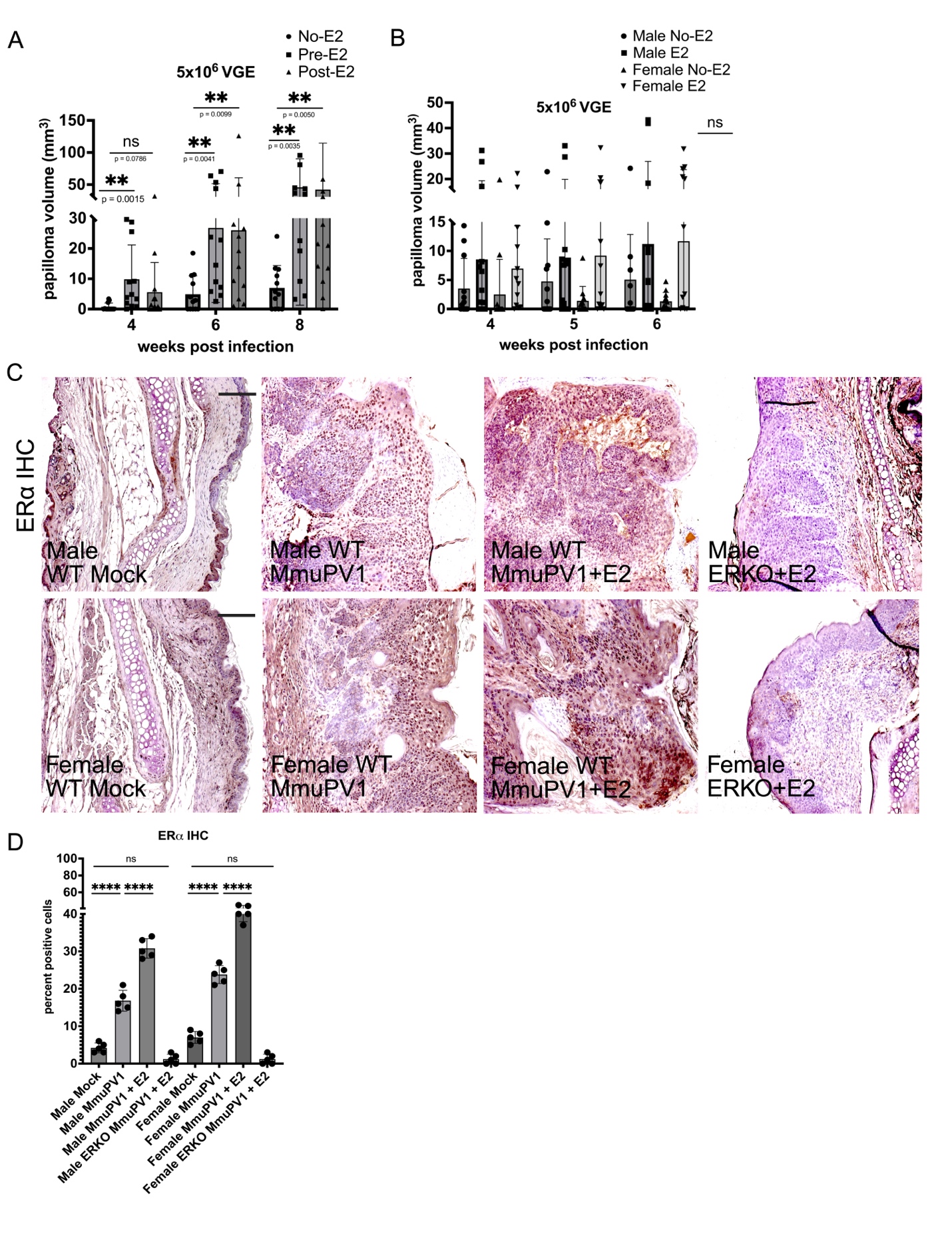
**

**Fig S1. Estrogen enhances papilloma growth independent of timing of administration**

**(A)** Papilloma volumes measured at 4, 6, and 8 weeks post‑infection in mice receiving no estradiol (E2), pre‑E2 treatment, or post‑E2 treatment. Data from male and female mice were combined to increase statistical power; sex‑specific results are shown in Fig. 1C. Both pre‑E2 and post‑E2 groups exhibited significantly increased papilloma growth over time compared with untreated controls, with statistical comparisons indicated. **(B)** Papilloma volumes at 4, 5, and 6 weeks post‑infection stratified by sex and E2 treatment status, demonstrating no significant sex‑dependent differences in wart growth. The grouped analysis corresponding to this experiment is presented in Fig. 1E. Data are shown as mean ± SD with individual data points displayed. Statistical significance was assessed using Student’s *t*‑test, Fisher’s exact test, and the Mann-Whitney U test (Wilcoxon rank‑sum test), as appropriate. **(C)** Representative ERα immunohistochemistry (IHC) images from ear skin of male and female WT and ERKO mice following mock treatment, MmuPV1 infection, or MmuPV1 infection with estradiol (E2) supplementation. Baseline ERα expression in mock‑treated epithelium was low in both sexes and associated with a thin, tightly organized epidermal layer. In contrast, MmuPV1 infection markedly increased epithelial ERα positivity, coinciding with pronounced virus‑induced epidermal hyperplasia. ERα immunoreactivity was further elevated in E2‑supplemented infected tissues, which displayed even greater epidermal thickening. This pattern was observed in both males and females, although females exhibited higher overall ERα positivity. As expected, ERKO mice showed no ERα staining, confirming loss of receptor expression. **(D)** Quantification of ERα‑positive epithelial cells expressed as percentage of positive cells per field (5 fields per wart). Data are presented as mean ± SEM. Scale bars = 100μm. ns = not significant; *p < 0.05; **p < 0.005; ***p < 0.01; ****p < 0.001.

**
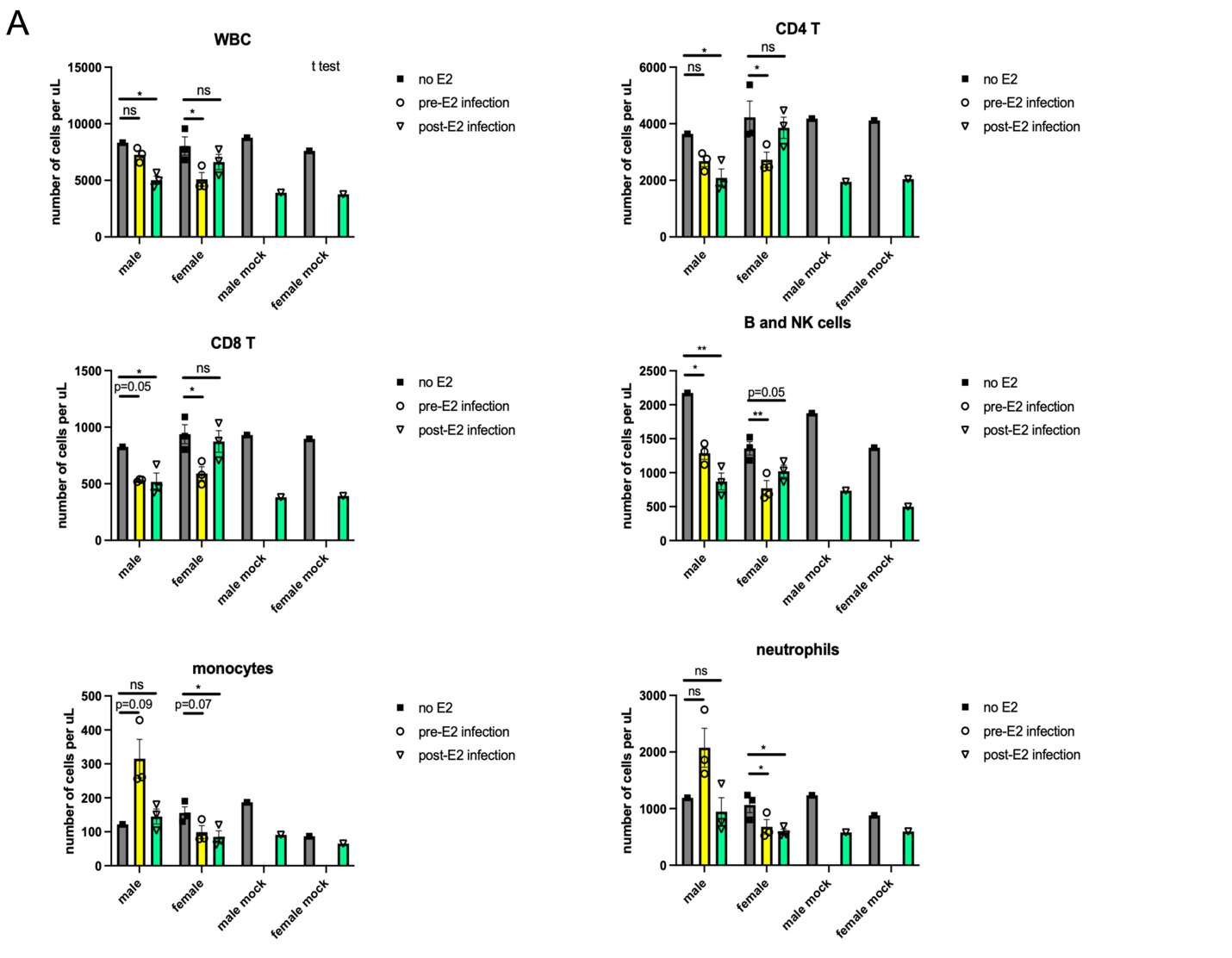
**

**Fig S2. Estrogen reduces a subset of peripheral immune cell counts during MmuPV1 infection.**

**(A)** Quantification of major immune cell populations, including total white blood cells (WBC), CD4⁺ T cells, CD8⁺ T cells, B and NK cells, monocytes, and neutrophils. Comparisons were made among groups receiving no E2, pre-E2, or post-E2 treatment. Estrogen reduces a subset of circulating immune cells in both sexes; however, this effect is not significant when analyzed separately by sex. Grouped analysis for these data is shown in Figure 2C. Data are presented as mean ± SD with individual data points displayed. ns: not significant. *p < 0.05, **p < 0.005, ***p < 0.001, ****p < 0.0001.


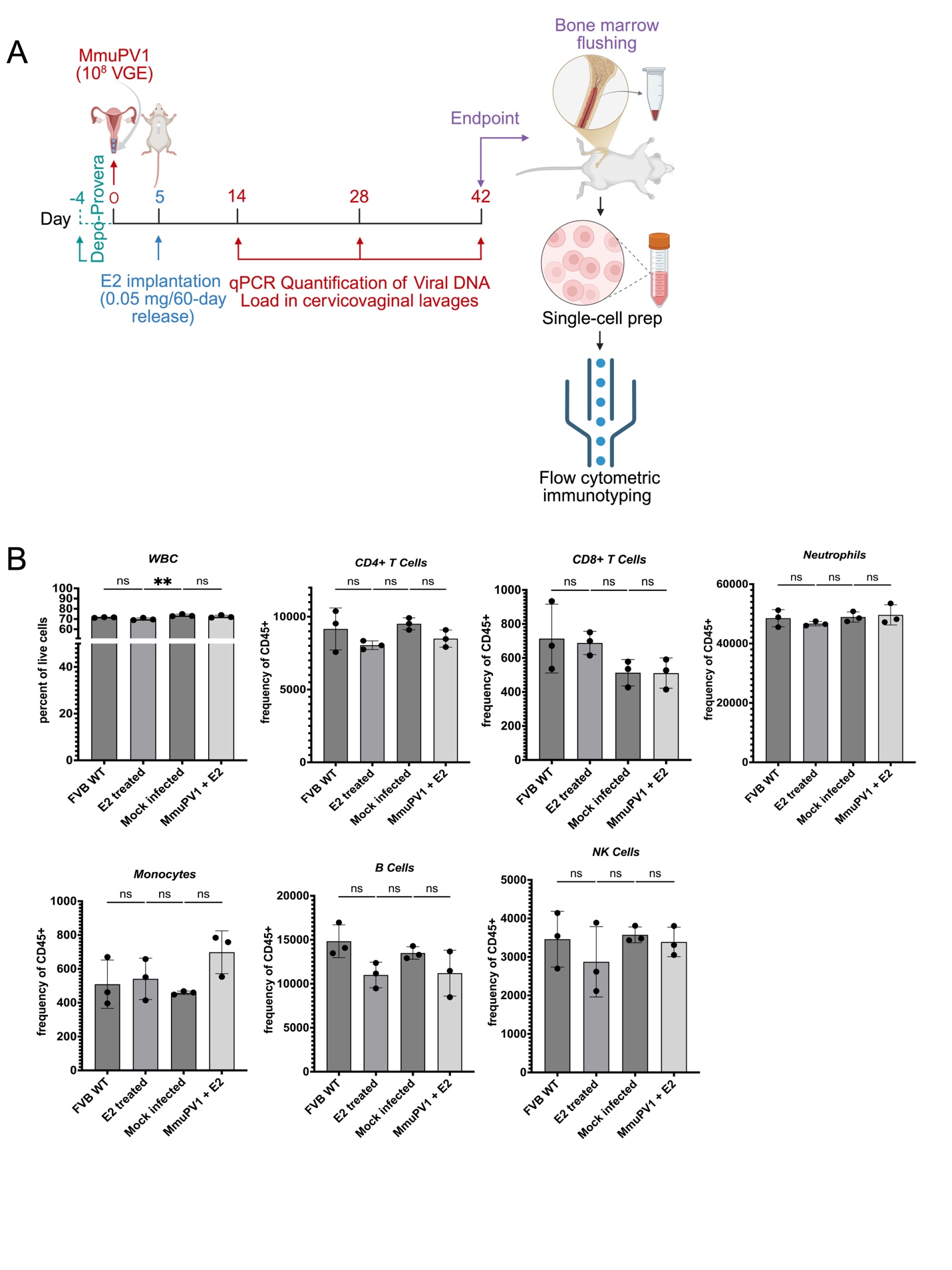


**Fig S3. Estrogen does not modulate hematopoietic immune cell output in the context of MmuPV1 infection.**

**(A)** Schematic diagram created in BioRender (<https://BioRender.com/bf7209s>) illustrating the MmuPV1 cervicovaginal infection model in FVB mice. At 8 weeks post‑infection (8 wpi), mice were euthanized and bone marrow was harvested by flushing two tibias and two femurs per mouse. Single‑cell suspensions were prepared for multiparameter flow cytometric immunophenotyping to determine whether the E2 -induced systemic reductions in circulating immune cells observed both in our previous work and in the current study could be explained by sequestration or redistribution of immune populations within the bone marrow compartment. **(B)** Flow cytometric analysis revealed no significant enrichment of any major immune subset including total CD45⁺ leukocytes, CD4⁺ T cells, CD8⁺ T cells, neutrophils, monocytes, B cells, or NK cells in bone marrow from E2‑treated or MmuPV1‑infected animals compared with E2‑only or mock‑infected controls. Although modest increases in neutrophils and monocytes were observed, these changes were not statistically significant. Importantly, estradiol alone did not alter bone marrow immune composition (Fig S3A-B), Together, these findings suggest that the previously reported estradiol‑mediated depletion of circulating immune cells as well as the reductions observed in the current study is not explained by immune cell sequestration within the bone marrow, but instead likely arises from alternative mechanisms that will require future investigation. Data are presented as mean ± SD with individual data points displayed. ns: not significant. *p < 0.05, **p < 0.005, ***p < 0.001, ****p < 0.0001


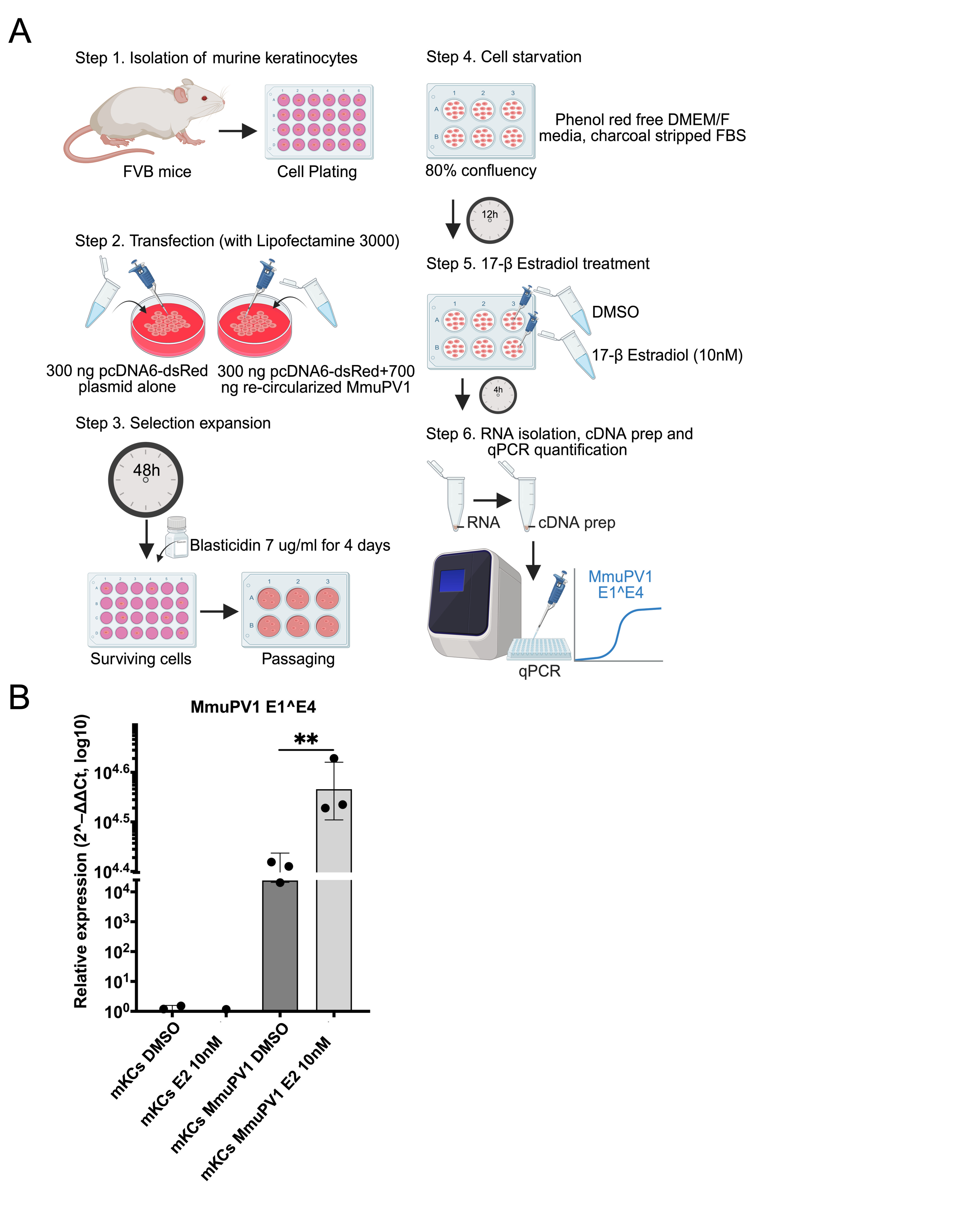


**Figure S4. Estrogen directly enhances MmuPV1 early gene expression in keratinocytes harboring re‑circularized viral genomes**

**(A)** Schematic of experimental workflow for transfection of mouse keratinocytes with MmuPV1 re-circularized genomes. Illustration created with BioRender (<https://BioRender.com/l7pwgfr>). Primary keratinocytes isolated from FVB mice were plated and transfected with either (i) a pcDNA6‑dsRed plasmid alone (300 ng) or (ii) re‑circularized MmuPV1 genomes (700 ng) together with pcDNA6‑dsRed (300 ng) using Lipofectamine 3000. After 48 hours, cells underwent blasticidin selection (7 µg/mL, 4 days) and were subsequently expanded. Once cultures reached ~80% confluence, cells were starved overnight in phenol-red-free DMEM/F medium supplemented with charcoal‑stripped serum. The following day, cells were treated for 4 hours with either DMSO vehicle control or 17β‑estradiol (10 nM). RNA was isolated, converted to cDNA, and MmuPV1 early gene expression (E1^E4) was quantified by qPCR. **(B)** qPCR analysis of MmuPV1 E1^E4 transcript levels (relative expression plotted on a log₁₀ scale). Keratinocytes harboring re‑circularized MmuPV1 genomes exhibited a significant increase in E1^E4 expression following 17β‑estradiol treatment compared with DMSO control. No viral transcripts were detected in wild‑type keratinocytes, confirming assay specificity. Data are presented as mean ± SD with individual data points shown. ns, not significant*p < 0.05, **p < 0.005, ***p < 0.001, ****p < 0.0001

**
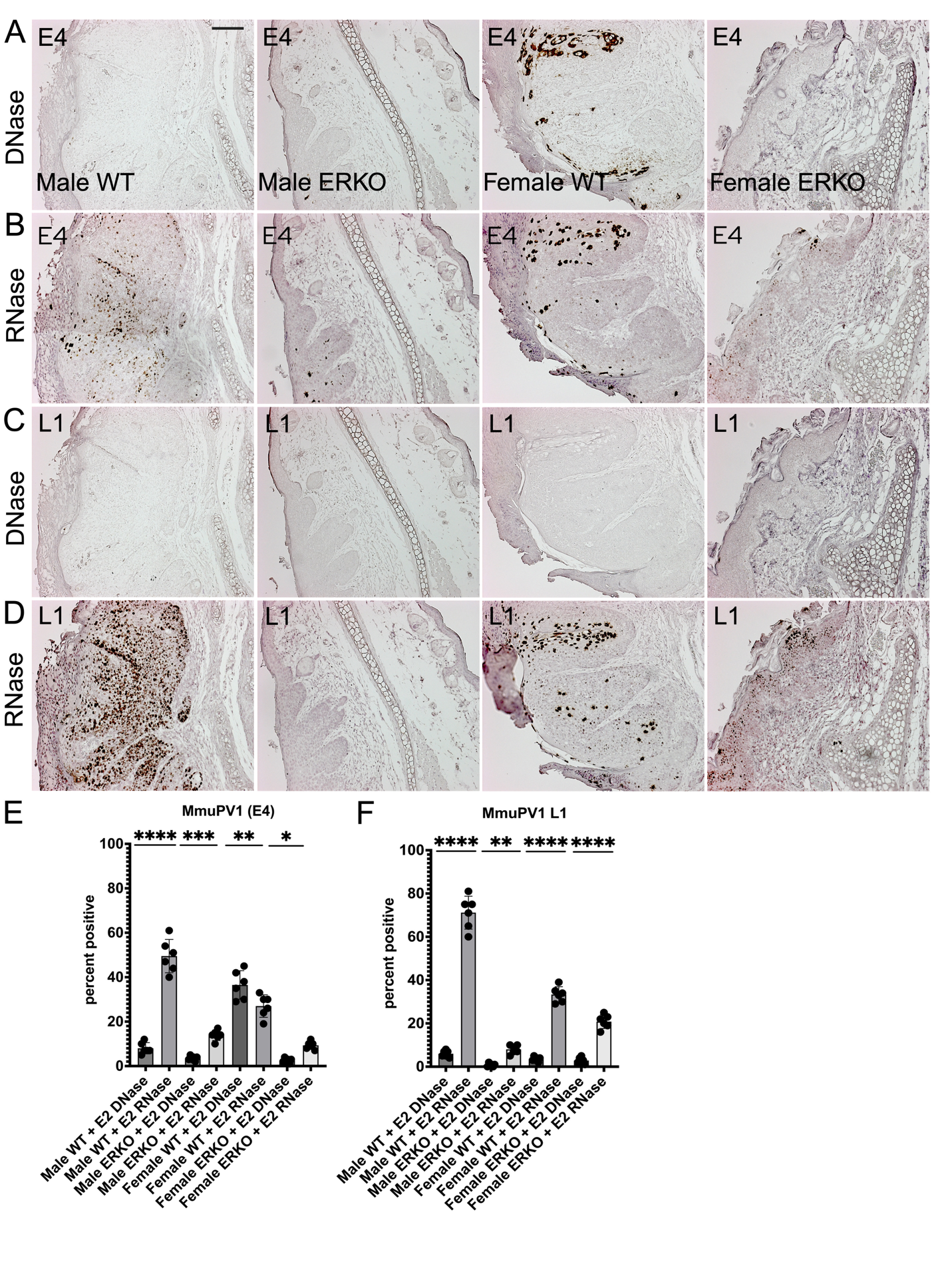
Fig S5. Nuclease digestion reveals that estradiol‑enhanced MmuPV1 E4 and L1 signals originate from viral DNA rather than RNA.**

**(A-D)** RNA in situ hybridization (RNAScope) for MmuPV1 E4 (A-B) and L1 (C-D) performed on ear lesions from male and female wild‑type (WT) and ERα‑deficient mice supplemented with estradiol. To determine whether estradiol‑mediated increases in viral signal derive from viral RNA or viral DNA, adjacent sections were pre‑treated with DNase (A, C) or RNase (B, D) prior to probe hybridization. Estradiol‑treated WT lesions retained strong E4 and L1 puncta following RNase digestion, whereas signal was abolished by DNase treatment, except for DNase-treated WT females demonstrating that the majority of detectable RNAscope signal reflects viral DNA rather than RNA transcripts. These results are consistent with elevated viral genome copy number under estradiol conditions and this interpretation aligns with prior qPCR‑based longitudinal studies from our laboratory showing increased viral genome equivalents in estradiol‑treated mice during cervicovaginal MmuPV1 infection. Estradiol markedly increased E4 and L1 signal intensity in WT mice of both sexes, whereas ERα‑deficient mice exhibited minimal to no detectable signal, confirming assay specificity and effective *Esr1* deletion. **(E–F)** Quantification of E4 (F) and L1 (G) signals demonstrates significantly higher viral DNA signal in WT mice compared with ERKO mice, confirming that *Esr1*‑mediated estrogen signaling is required for robust MmuPV1 replication. Data are presented as mean ± SEM. Scale bar = 100 µm. ns = not significant; *p < 0.05; **p < 0.005; ***p < 0.001; ****p < 0.0001.

**
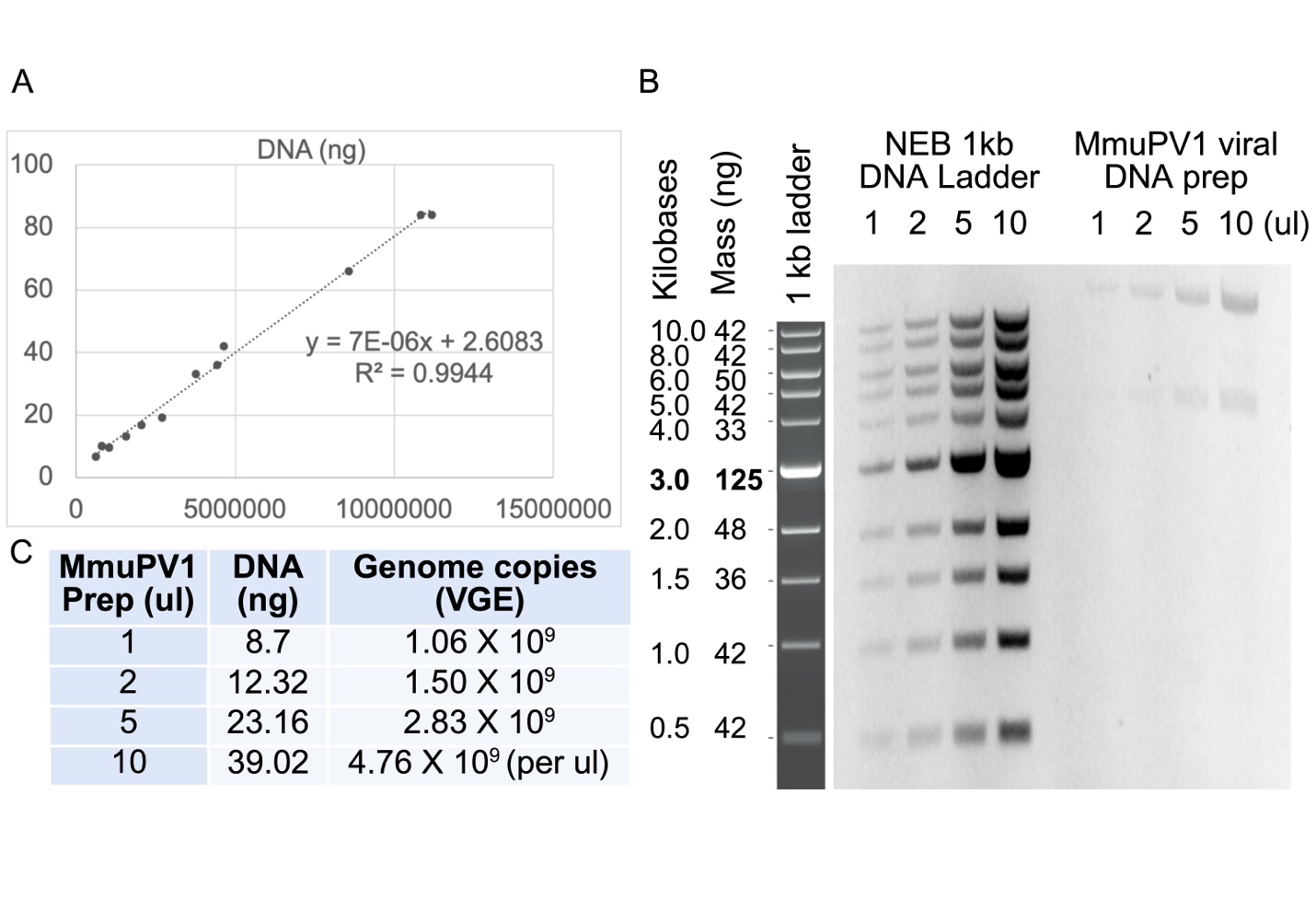
**

**Fig S6. Quantification of MmuPV1 viral genome equivalents (VGE) using NEB 1 kb DNA ladder-based standard curve analysis.**

**(A)**A standard curve was generated using band intensities from the NEB 1 kb DNA ladder plotted against the known DNA mass per band. Linear regression of the plotted values produced a highly correlated standard curve (R² = 0.9944), enabling interpolation of unknown DNA quantities from gel band density measurements. **(B)** A 1% agarose gel showing the NEB 1 kb DNA ladder loaded at 1, 2, 5, and 10 µL alongside MmuPV1 viral DNA preparations loaded at the same volumes. Ladder bands of known mass and viral DNA bands were resolved under identical electrophoretic conditions. **(C)** Band intensities from both the ladder and MmuPV1 viral prep lanes were quantified using Image Lab software. Well-resolved ladder bands were used to generate a regression‑based mass estimate for the viral DNA bands. The derived DNA mass values (ng) were then used to calculate MmuPV1 genome copies/ viral genome equivalents (VGE) used in the experiments.
